# A multipronged strategy of an anti-terminator protein to overcome Rho-dependent transcription termination

**DOI:** 10.1093/nar/gks872

**Published:** 2012-09-29

**Authors:** Ghazala Muteeb, Debashish Dey, Saurabh Mishra, Ranjan Sen

**Affiliations:** Laboratory of Transcription, Center for DNA Fingerprinting and Diagnostics, Tuljaguda Complex, 4-1-714 Mozamjahi Road, Nampally, Hyderabad 500 001, India

## Abstract

One of the important role of Rho-dependent transcription termination in bacteria is to prevent gene expressions from the bacteriophage DNA. The transcription anti-termination systems of the lambdoid phages have been designed to overcome this Rho action. The anti-terminator protein N has three interacting regions, which interact with the mRNA, with the NusA and with the RNA polymerase. Here, we show that N uses all these interaction modules to overcome the Rho action. N and Rho co-occupy their overlapping binding sites on the nascent RNA (the *nutR/tR1* site), and this configuration slows down the rate of ATP hydrolysis and the rate of RNA release by Rho from the elongation complex. N-RNA polymerase interaction is not too important for this Rho inactivation process near/at the *nutR* site. This interaction becomes essential when the elongation complex moves away from the *nutR* site. From the unusual NusA-dependence property of a Rho mutant E134K, a suppressor of N, we deduced that the N-NusA complex in the anti-termination machinery reduces the efficiency of Rho by removing NusA from the termination pathway. We propose that NusA-remodelling is also one of the mechanisms used by N to overcome the termination signals.

## INTRODUCTION

The factor-dependent transcription termination in bacteria is carried out by a homo-hexameric RNA-dependent ATPase, called Rho (1–3). In this termination process, Rho at first recognizes 70–80 nt of unstructured C-rich sequence known as rho utilization (*rut*) site on the nascent RNA (4,5) through its N-terminal primary RNA binding site (PBS; 6,7). This binding event guides the 3'-side of the RNA into the central hole of the hexamer, which constitutes the secondary RNA binding site. This in turn activates its ATP hydrolysis and translocase activity, and the latter function is believed to be instrumental in dislodging the elongation complex (EC; 8) inside a termination zone, which is usually located 60–90 nts downstream of the *rut* site (9).

It is envisioned that the Rho-dependent termination in bacteria has evolved not only to enforce a premature termination of RNA synthesis in case of the failure of ribosome-loading onto the mRNA but also to play a major role in preventing the deleterious effects of transcription of the foreign DNA injected by the bacteriophages (3). The anti-termination strategies of the bacteriophages were primarily designed to combat Rho-dependent termination process (10,11). In general, these strategies involve the modifications of the EC by phage-coded factors (protein or RNA) in such a way that it can pass through the terminator signals without getting dislodged from the template DNA.

**Figure 1.**
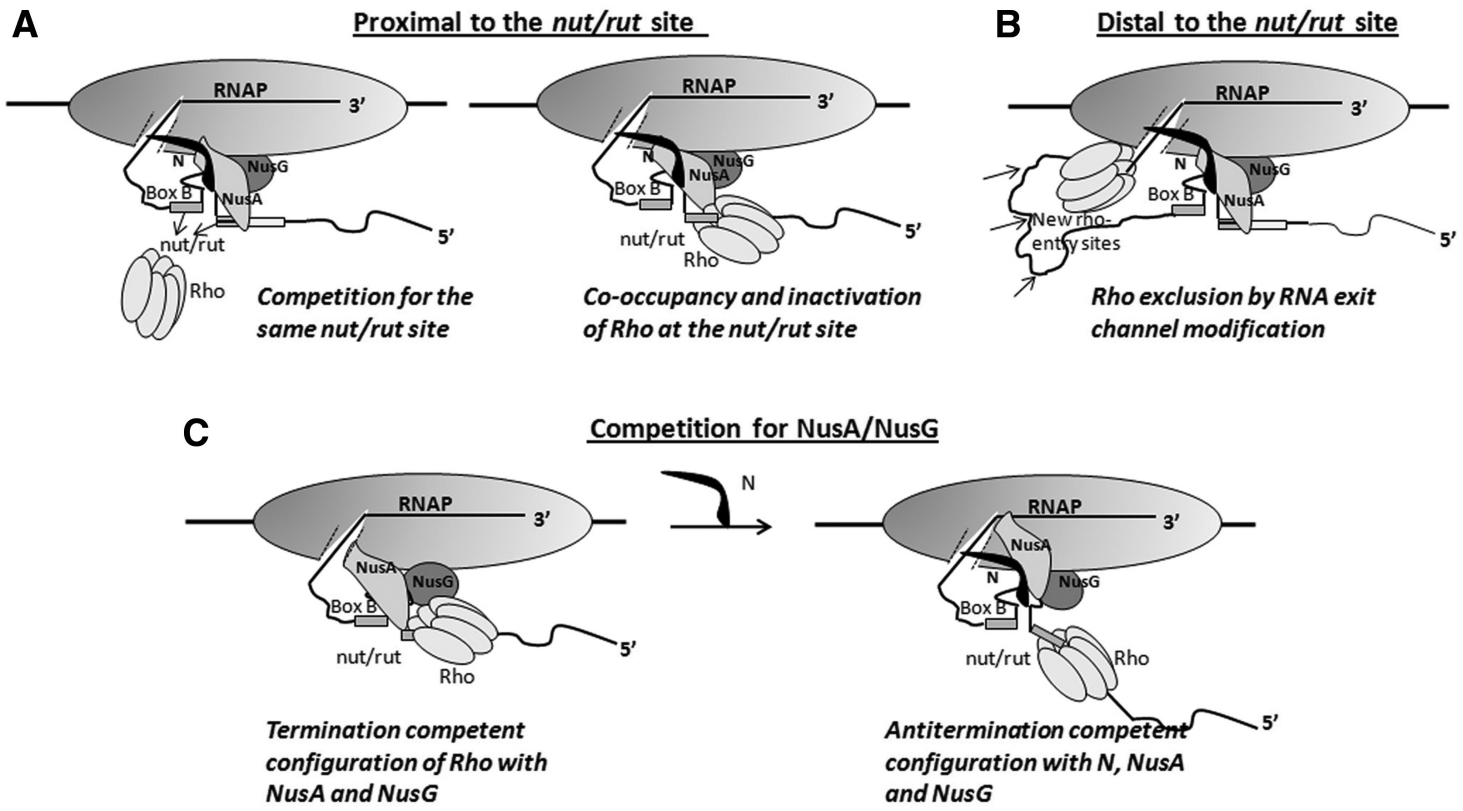
Cartoons showing the possible hypotheses for overcoming Rho-dependent termination by N. (**A**) When the EC is near the *nut/rut* site, Rho action can be inhibited by N either by a direct competition mechanism for the same site on the nascent RNA (left panel) or N and Rho can co-occupy the same site, and this configuration delays the Rho activation step(s) (ring-closure and initiation of ATP hydrolysis; right panel). (**B**) When the EC moves away from the *nut/rut* site, Rho can be excluded by N modification of the RNA exit channel through which Rho is likely to approach the RNAP. (**C**) N functionally removes NusA and NusG from the Rho-dependent termination pathway by remodelling the interactions.

N protein coded by the lambdoid phages is a well-known anti-terminator that modifies the host RNA polymerase (RNAP) during the transcription elongation process together with the Nus factors (NusA, NusG, NusB and NusE) of the host transcription machinery. This modification helps the EC to express the middle and late genes of lambdoid phages by suppressing many Rho-dependent and -independent terminators present on the phage DNA (10,11). N is a small RNA-binding protein that interacts with a RNA-hairpin structure (*boxB*) present in the N utilization (*nut*) site of the nascent RNA through its N-terminal arginine rich motif (ARM; Supplementary Figure S1A and B; 12). The central domain of N interacts with NusA (13) and recruits the latter to the *spacer* region of the *nut* site (14), and subsequently this N-NusA-*Nut* RNA complex works as a platform to recruit other Nus factors (15; also see the cartoons in Figure 1). The C-terminal regions of N binds to the RNAP (13) near the RNA exit channel of the latter (16), which may involve penetration of part of this region of N into the active centre of the EC (17). This configuration of N-Nus-EC complex makes the transcription elongation process on the phage DNA highly processive over a long distance (10).

The mechanism of N-mediated suppression of RNA hairpin-dependent termination has been studied in detail (16,18,19). However, the mechanism of anti-termination of the Rho-dependent termination by N is not known. In this report, we have provided genetic and biochemical evidence for a multipronged strategy used by N to overcome the Rho function. We showed that N (i) inactivates Rho at the *nutR* site by forming a N-NusA-Rho ternary complex, which renders slow rate of ATP hydrolysis of the former; (ii) exerts anti-termination most likely by modifying the RNA exit channel of the EC, which is operational even far away from the *nut* site; and finally (iii) removes NusA from the Rho-dependent termination path.

## MATERIALS AND METHODS

### Bacterial strains, phages and plasmids

Bacterial strains, plasmids and phages used in this study are listed in Supplementary Table S4. All the *in vivo* anti-termination assays were performed in different derivatives of *Escherichia coli* rac*^−^* strain MC4100. The strains GJ5147 and RS445 used in β-galactosidase assays contain single-copy P*_lac_*-H-19B *nutR/tR1- lacZYA* (GJ5147) or P*_lac_-lacZYA* (RS445) reporter cassettes as λRS45 lysogen. Strain RS1017 was constructed by moving P*_lac_–* H-19B *nutR/tR1- trpt'-lac ZYA* reporter cassette by λRS45 mediated transduction from pRS992 into the strain RS257. This construct has two terminators *tR1* and *trpt'* attached sequentially. Strain RS1018 and RS1019 were also constructed by moving P*_lac_*-H-19B *nutR/tR1-tR’-lacZYA* and P*_lac_-*λ*nutR/λtR1–lacZYA,* respectively*,* in the same way into RS257. Temperature-sensitive (ts) allele of *rpoC* [*rpoCR120* (ts)] was moved to RS445, RS734 and RS1017, resulting in strains RS940, RS941and RS1029, respectively, by P1 transduction. Plasmid pRS1092 was constructed by inserting *trpt'* sequence at the SmaI site present after the *nutR/tR1* sequence of pRS22 (pTL61T with pT7A1– H-19B *nutR-tR’*-T1T2-*lacZYA*) to make the double terminator, *tR1-trpt'*, construct. This *trpt'* sequence was amplified from pRS992 using oligos RS567/RS568. XL-red strain (Stratagene) was used for random mutagenesis (20). The mutagenesis procedure is described in the supplementary methods.

### Measurement of *in vivo* anti-termination

β-galactosidase activities from the *lacZYA* reporter is fused to different terminator constructs were used to measure the *in vivo* anti-termination. Ratios of −β-galactosidase activities obtained in the presence and absence of terminator cassettes gave the measure of anti-termination (% RT). The strains RS734 and RS445 were transformed with the plasmids having the mutants and wild-type (WT) H-19B N genes to estimate the *in vivo* anti-termination efficiency at the H-19B *tR1* rho-dependent terminator (Table 1). Similarly, the strains RS1017 and RS1018 were transformed with these plasmids to get the β−galactosidase activities at H-19B *tR1-trpt’* terminators (Table 1) and at intrinsic terminator *tR’* (Supplementary Table S1), respectively. The strains RS1019 and RS445 were transformed with the plasmids having mutant and WT λ N genes to estimate the *in vivo* anti-termination efficiency at λ*tR1 rho*-dependent terminator (Table 1). The strains RS940, RS941 and RS1029 were used to get the β-galactosidase activities in the presence of mutant RNAP, carrying the *ts* alleles of *rpoCR120* (*rpoC*). In this case, strains were grown at 42°C, to inactivate the chromosomal copy of the WT *rpoC* gene (Table 1).

**Table 1.** *In vivo* antitermination at different Rho-dependent terminators by various N and RNAP alleles

Source of N	N alleles	RNAP alleles	P*_lac_*-*nutR/t_R1_*-*lacZYA*^a^			P*_lac_*-*nutR/t_R1_-trpt'-lacZYA*^b^		
			β−galactosidase activities (A.U.)			β−galactosidase activities (A.U.)		
			+ter	−ter	%RT	+ter	−ter	%RT
H-19B	WT	WT	998 ± 25	2916 ± 89	34.2	927 ± 67	2916 ± 89	31.8
	R3H		335 ± 17	4196 ± 554	7.9			
	S11F		223 ± 16	4404 ± 565	5.1			
	R15C		136 ± 12	3374 ± 331	4.0			
	R15P		142 ± 9	3844 ± 335	3.7			
	R18P		157 ± 4	3545 ± 709	4.4	3.9 ± 1.2	2201 ± 150	0.18
	Δ78-127		293 ± 45	3574 ± 488	8.2			
	Δ88-127		445 ± 21	3611 ± 590	12.3			
	Δ96-127		489 ± 11	2745 ± 372	17.8	38 ± 2	2745 ± 372	1.4
	Δ101-127		730 ± 14	2485 ± 152	29.4	243 ± 16	2485 ± 153	9.8
	Δ106-127		670 ± 21	3006 ± 116	22.3	368 ± 25	3006 ± 116	12.2
	Δ111-127		648 ± 17	2851 ± 112	22.7	166 ± 8	2851 ± 112	5.8
	Δ121-127		769 ± 11	2912 ± 130	26.4	707 ± 36	2912 ± 130	24.3
λ^c^	WT	WT	1026 ± 70	2699 ± 196	38.0			
	Δ73-107		192 ± 13	2642 ± 175	7.3			
	Δ81-107		860 ± 65	2450 ± 292	35.1			
	Δ91-107		940 ± 64	2613 ± 210	36.0			
	Δ101-107		989 ± 50	2488 ± 139	39.8			
H-19B^d^	WT	WT	980 ± 25	1969 ± 82	49.7	834 ± 31	2049 ± 90	40.7
		P251S, P254L	460 ± 41	1327 ± 45	34.7	303 ± 24	1565 ± 102	19.3
		R270C	883 ± 33	2934 ± 342	30.1	636 ± 35	2573 ± 262	24.7

The above strains were transformed with the plasmids bearing different WT and mutant H-19B N (or λ N) genes. The ratio of β-galactosidase values in the presence (+ter) and absence (−ter) of terminator gives the efficiency of terminator read-through (%RT). Two terminator-*lacZYA* fusions, *t_R1_-lacZYA* and *t_R1_-trpt'-lacZYA*, were used. The Rho-dependent terminator, t_R1_ was derived from the *nutR-cro* region of either a lambdoid phage H-19B (for H-19B N) or the λphage (for λ N). The errors are calculated from the average of 4 to 5 independent measurements.

^a^Strains RS734 (+ter) and RS445(−ter)

^b^Strains RS1017(+ter) and RS445(−ter)

^c^Strains RS1019 and RS445 with *nutR/tR1* of λ-phage

^d^Strains RS941(+ter) and RS940(−ter); ^e^Strains RS1029 (+ter) and RS940 (−ter); Experiments were performed at 42°C to inactivate the temperature sensitive (*ts*) allele of WT *rpoC* present in the chromosome and the WT and mutant *rpoC* were supplied from the plasmids. Anti-termination efficiency increases at higher temperature. These two *rpoC* mutants did not support H-19B N mediated anti-termination and also the growths of λ and H-19B phages (16). These mutants are located near the RNA exit channel of the EC.

To estimate the *in vivo* termination efficiency of the suppressor mutants of Rho at the H-19B tR1- *trpt'* terminator, the strains RS1017 and RS445 containing a plasmid bearing H-19B N gene (pK8601) were transformed with the suppressor and the WT Rho plasmids, and were subsequently made Δrho in the chromosome by P1 transduction (Figure 6A). Ratios of the β-galactosidase activities from the lysogens present in RS1017 and RS445 gave the measure of the termination efficiency. Termination defects of these suppressor mutants were also measured in the absence of WT H-19B N (devoid of pK8601; Supplementary Table S2).

All the measurements of β-galactosidase activities were done in a microtiter plate using a Spectramax plus plate reader following the published procedure (21,22).

### *In vitro* transcription assays

**Figure 2.**
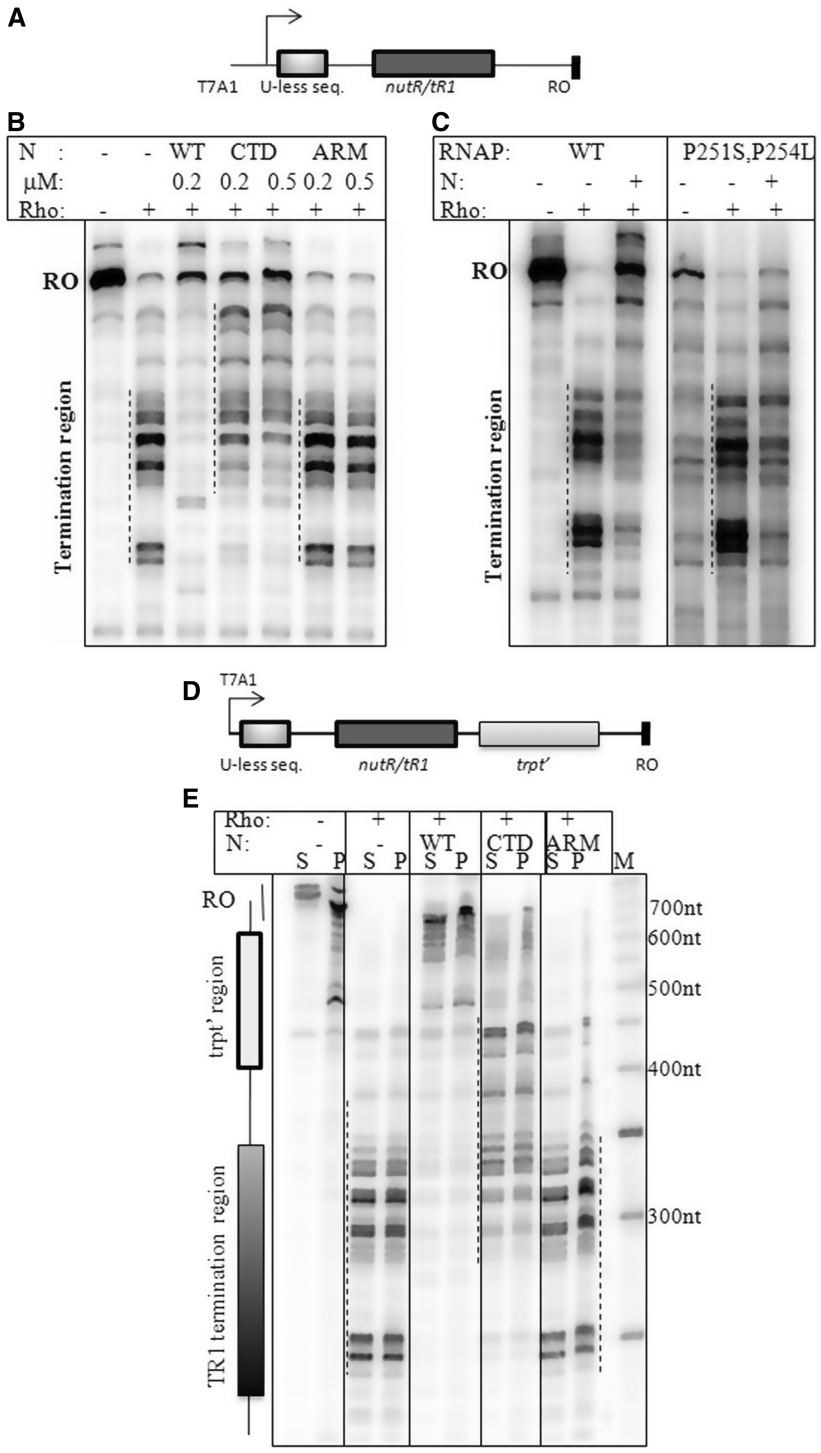
*In vitro* transcription termination assays on H-19B *nutR/tR1*and H-19B *nutR/tR1*- *trpt'* terminator templates. (**A**) Cartoon of the H-19B *nutR/tR1* DNA template. Autoradiograms showing the single round *in vitro* transcription termination in the presence of different derivatives of N (**B**) or RNAP (**C**). Termination regions are indicated by dotted lines next to the transcript bands. RO denotes the run-off product. The concentration of Rho was 50 nM and that of N as indicated in (B) and 200 nM in (C). Amounts of WT RNAP holoenzyme and RNAP mutant were 25 nM and 50 nM, respectively. In all, 100 nM σ^70^ was added to the RNAP mutant in (B). (**D**) Cartoon showing the design of the double terminator template. (**E**) Autoradiogram showing the single round *in vitro* transcription termination in the presence of WT/mutant H-19B N on the immobilized template. Lanes denoted as ‘S’ indicate half of the supernatant, and ‘P’ denotes the rest of the reaction mix. ‘RO’ denotes the run-off product. The concentrations of Rho and N were 50 nM and 200 nM, respectively. Released RNA will be in the ‘S’ lanes. Termination zones are indicated by dotted lines and on the left side of the gel.

**Figure 3.**
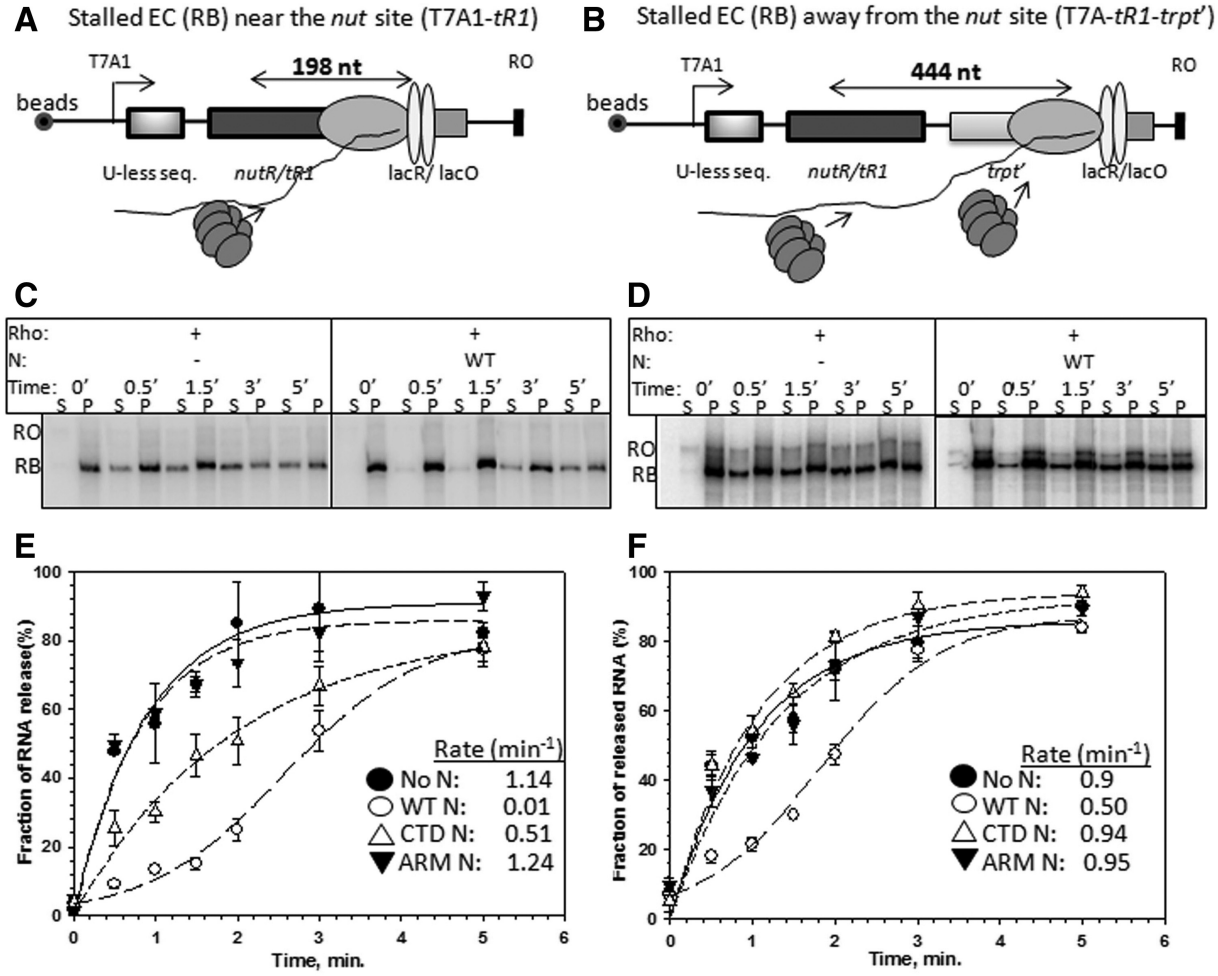
Effect of N on the Rho-mediated RNA release from the stalled elongation complexes at different distances from the *nut* site. Cartoon showing the designs of stalled elongations complexes (RB) near the H-19B *nutR*/*tR1* single terminator region (**A**) and after the H-19B *nutR/tR1-trpt'* double terminator region (**B**) using *lac* repressor as a road-block*.* Distances from *nutR-boxB* to *lacO* sites in both the templates are indicated. Autoradiograms showing the amount of RNA released by Rho, in the absence and presence of WT H-19B N at different time points from the RBs made on the single terminator (**C**) and the double terminators templates (**D**). ‘RO’ denotes the RO products formed from the ECs that reached the end of the template. Concentrations of Rho and H-19B N were 50 nM and 100 nM, respectively. These two templates were immobilized on streptavidin-coated magnetic beads. The 0' time points were obtained from incubating the RB in buffer having no Rho protein. ‘S’ denotes half of the supernatant, and ‘P’ denotes the rest of the sample. Fractions of RNA release was estimated as, [2S]/([S] + [P]) and were plotted against time (**E**, single terminator and **F**, double terminator) in the absence and presence of WT H19B N and its derivatives*.* Error bars are calculated from 2 to 3 independent measurements. The rates of RNA release indicated in the panels were calculated from the curve using the exponential rise equations. In case of +WT N curves, the rates were obtained from the initial slope.

*In vitro* Rho-dependent termination reactions were performed in T buffer (25 mM Tris–HCl (pH 8.0), 5 mM MgCl_2_, 50 mM KCl, 1 mM DTT and 0.1 mg/ml of BSA) at 32°C. The reactions were initiated with 10 nM DNA, 40 nM WT RNAP, 175 μM ApU, 5 μM each of GTP and ATP and 2.5 μM CTP to make a 23-mer EC_23_. [α- ^32^P]CTP (3000 Ci/mmol) was added to the reaction to label the EC_23_. The complex was chased with 250 μM NTPs in the presence of 10 μg/ml of rifampicin for 15 min at 32°C. Also, 50 nM WT Rho, 200 nM NusG, 300 nM NusA and 100 nM WT or mutant H-19B N were added to the chase solution as indicated. The reaction products were separated on 8% sequencing gels and analysed by phosphorimager (Figures 2, 6 and 7). Transcription reactions with T7A1-*nutR–tR1-lacO* or T7A1-*nutR-tR1-trpt'-lacO* templates (Figure 3) were done under the same conditions described earlier. In both the cases, DNA was immobilized on the streptavidin-coated magnetic beads, and 100 nM lac repressor was added before chasing the 23-mer EC. For RNA release assays, reactions were chased for 2 minutes, washed once, followed by the addition of Rho in the presence of 1 mM ATP. The reaction was incubated at 32°C for different time points, and half of the supernatant was taken out for the ‘S’ lanes, and the rest was phenol extracted and used for the ‘S + P’ lanes (Figures 3 and 6; Supplementary Figure S4). Preparations of N, NusA and Rho proteins and the DNA templates are described in the supplementary methods.

### ATPase assays

**Figure 4.**
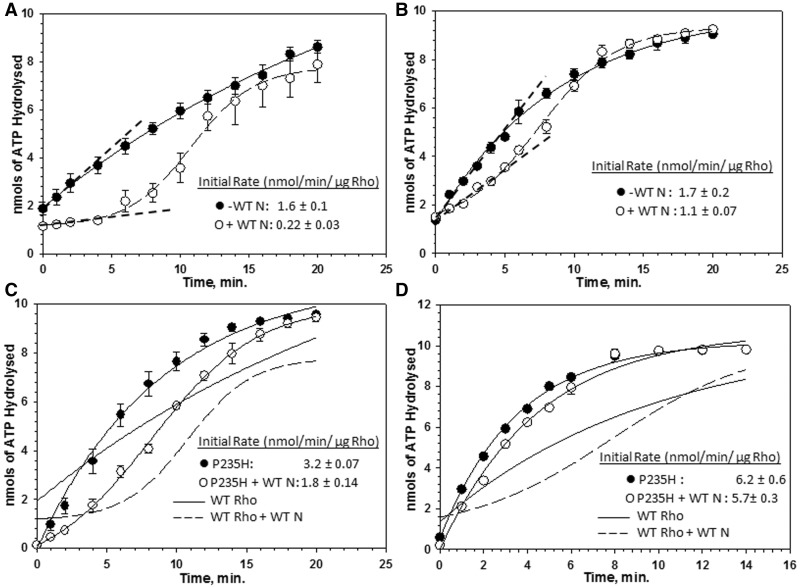
Effect of N on the rate of ATP hydrolysis by Rho. Amounts of [γ-^32^P]ATP hydrolysed by Rho in nanomoles are plotted against time both in the absence and presence of WT H-19B N. These assays were performed on the nascent RNA coming out of the stalled ECs formed (**A**) on single terminator (as in Figure 3A) and (**B**) on double terminator (as in Figure 3B) templates.100 nM each of Rho and H-19B N were used. In all, 300 nM NusA and 200 nM NusG were also present in these assays. The initial rates of ATP hydrolysis are indicated by dashed lines. Same experiments performed on ECs stalled at the single terminator (**C**) or double terminator template (**D**) (as in Figure 3) using P235H Rho. Rates of ATP hydrolysis by WT Rho are indicated by solid/dotted lines in (C) and (D). The rate values are stated in the panels.

ATPase activity of the WT Rho protein was measured from the release of inorganic phosphate (Pi) from ATP after separating on the polyethyleneimine (PEI)-cellulose TLC plates (Merck) with 0.75 M KH_2_PO_4_ (pH 3.5) as the mobile phase. In all the assays, the composition of the reaction mixture was 25 mM Tris–HCl (pH 8.0), 50 mM KCl and 5 mM MgCl_2_, 1 mM DTT and 0.1 mg/ml of BSA. Assays were performed on the nascent RNA emerging out of the transcription EC. Stalled ECs were formed at the lac operator site on the T7A1-*nutR/tR1-lacO* or T7A1-*nutR/tR1-trpt'-lacO* templates in the same way as described earlier. These complexes were incubated with 100 nM Rho in presence of 1 mM NTPs and [γ-^32^P]ATP (3000 Ci/mmol). Aliquots were removed and mixed with 1.5 M formic acid at various time points to stop the reaction. Release of Pi was analysed by exposing the TLC sheets to a Phosphorimager screen for 5 min and subsequently by scanning using Typhoon 9200 (Amersham), and the intensities of ATP and Pi were quantified by Image QuantTL software (Figure 4; Supplementary Figure S2 and S3).

Amounts of ATP hydrolysed (in nanomoles) were plotted against time using SIGMAPLOT software. The data points for all the curves, except for +N, were fitted to the equation of an exponential curve, y = a*[1-exp(-λ*x)]. The data points obtained for ‘+N’ experiments values were fitted to a sigmoidal equation; y = y_0_ + a/[1 + exp {-(x − x_0_)/λ}]. In these equations, ‘λ’ denotes the rate and ‘a’ is the amplitude. The r^2^ values for each of the fittings were ≥0.99. For rate calculations, only the initial slopes were considered as later points in the curves originated from multiple rounds of ATP hydrolysis by Rho after dislodging the EC. The initial slopes of the plots give ATPase activity of Rho in terms of nanomoles of ATP hydrolysed per minute (8,21).

For the ATPase assays of E134K using poly(C), 50 nM Rho was incubated with 1 mM ATP, together with [γ-32P]ATP (3500 Ci/mmol; BRIT, India) at 37°C, and ATP hydrolysis was initiated by the addition of 20 µM poly(C). Products were analysed by the same way as described earlier. The initial rates of the reaction were determined by plotting the amount of hydrolysed ATP versus time using linear regression method (Figure 7D).

### RNA footprinting

**Figure 5.**
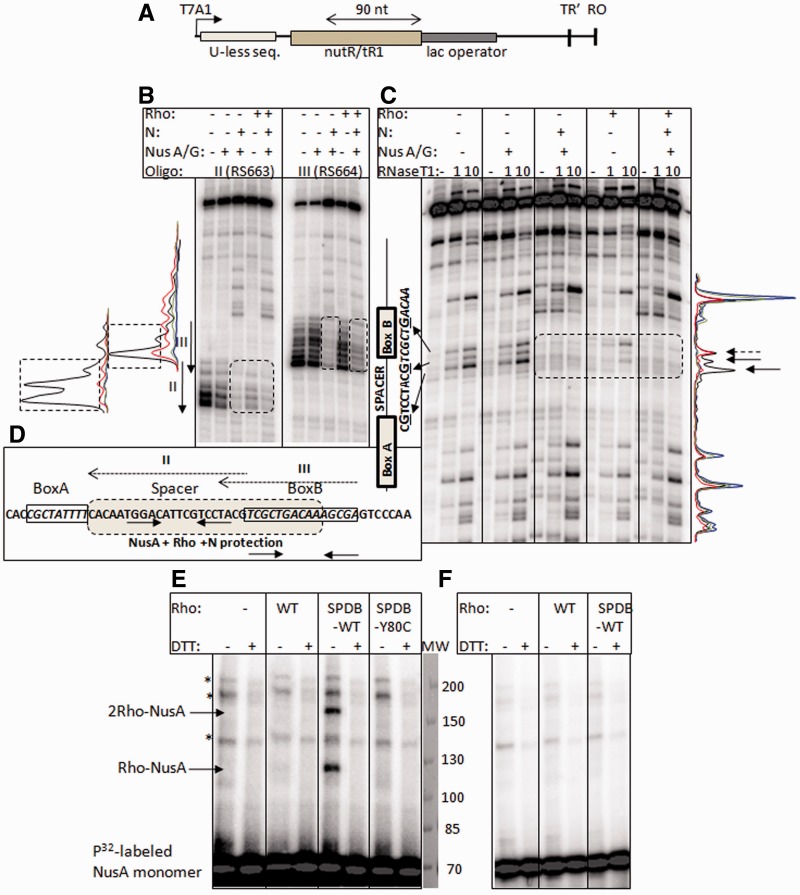
Co-occupancy of Rho and N together at the *nutR/tR1* site of the nascent RNA. (**A**) Cartoon showing the design of a RB complex at the H-19B *nutR*/*tR1* terminator sequence*.* Nascent RNA emerging out of this stalled EC was foot-printed using RNase H and RNase T1 under different conditions. The nascent RNA was effectively labelled only at the 5′-end by selectively incorporating [α−P^32^]CTP in the EC_23_ and chasing it to the lac operator site with excess cold NTPs. The distance from *boxB* to *lacO* site is indicated. (**B**) RNase H and (**C**) RNase T1 footprinting of the nascent RNA of the stalled EC (RB) under different conditions as indicated above the auto-radiograms. The locations of anti-sense oligos used for RNase H footprinting are indicated by arrows. The locations of RNase H and T1 cleaved sites on the RNA are indicated in between the two gels. Protections on RNA by Rho, N+ NusA/G or Rho + N + NusA/G are indicated by dotted boxes. The protected G residues in *spacer* and *boxB* regions are also indicated. The band intensity profiles are shown adjacent to the autoradiograms. Colour coding of the curves obtained from RNAse H and T1 cleavges are as follows: black, no factor; red, only Rho; blue, N +NusA/G; green, N, NusA/G, Rho. Protected area on these profiles are indicated either by dotted boxes (for RNAse H) or by arrows (for T1). (**D**) Protection of the *spacer* and *boxB* regions by NusA, Rho and N is indicated by a shaded box. The locations of anti-sense oligos used for RNase H footprinting are indicated by dotted arrows above the sequence. Hairpin structures are shown by solid arrows beneath the sequence. (**E**) Auto-radiograms of radio-labelled NusA on a non-reducing SDS-PAGE. Cross-linking reactions were performed in the presence of either unlabelled (WT) or SPDP labelled WT Rho (SPDP-WT) or SPDP-labelled Y80C Rho on the elongation complex (left panel; on the same stalled EC described in A). In the right panel (**F**), same experiments were performed by omitting RNAP from the reaction mix. The two cross-linked species are indicated, and their compositions were identified according to their molecular weights. The lane for molecular weight markers was stained with coomassie blue. Bands corresponding to the ‘*’ were non-specific in nature.

Footprinting assays were performed essentially in the same way as described in (23). For RNase H footprinting assays, four different DNA oligos, RS662, RS663, RS664 and RS665, antisense to H-19B *nutR boxA*, *spacer*, *boxB* and the region immediately after *boxB*, respectively, were used. Stalled EC was formed at the lacO site of the template pT7A1-*nutR*-lacO-*tR’* immobilized to the magnetic beads, in the same way as described earlier, in the presence of N, NusA and NusG. The EC was washed to remove free NTPs and was then incubated with 50 nM Rho in presence of 1 mM AMPPNP. In all, 10 μM of each of the anti-sense oligos were then added to the reactions for 30 sec, following which one unit RNase H was added and incubated for 1 min at 32°C. The reaction was stopped by extracting with phenol, mixed with equal volume of form amide loading dye and loaded onto an 8% sequencing gel (Figure 5B).

For RNase T1 footprinting, stalled EC at the terminator was formed in a similar way on the same template as described for RNase H foot printing. Stalled EC was washed extensively before footprinting, followed by incubation for 5 min with 50 nM WT Rho in presence of 1 mM AMPPNP. In all, 1U or 10U of T1 was added, and the reaction was performed for 1 min at 32°C. Reactions were stopped by phenol extraction. In both the footprinting assays, the region of nut site was identified from the anti-sense oligo-binding sites, migration of DNA markers and the T1 sensitive single-stranded G residues (Figure 5C).

### Cross-linking of Rho and NusA

We have used a bi-functional cross-linker, LC-SPDP (sulfosuccinimidyl 6-[3′(2-pyridyldithio)-propionamido] hexanoate (Pierce), which cross-links cysteine and amine. We first labelled the surface-exposed primary amines (from lys residues) of a no-cysteine derivative of WT (C202A) and Y80CRho (C202A). The mutant Y80C Rho could not be made ‘zero’ cys to maintain the Y80C mutation. This derivative of Rho has 28 lys residues against one cys (at the position 80) per monomer; therefore, on SPDP labelling, majority of the modifications will be in the lysines, and it is likely that cysteins will not be labelled. The concentrations of the cross-linker and Rho were 5 μM and ∼18 μM (monomer concentration, which is also the concentration of the cysteines), respectively. Concentration of the cross-linker was kept less to get a sub-saturating labelling of Rho, which ensured the absence of non-specific cross-linking in the subsequent steps. The lowest concentration of SPDP was determined by trials. Rho and SPDP were mixed in phosphate buffer (100 mM NaH_2_PO4, 150 mM NaCl, 1 mM EDTA, pH 7.5), and incubation was continued for 30 min at 25°C. Excess SPDP was removed by passing the mixture through a protein-desalting column (Pierce). In all, 100 nM of SPDP-derivatized Rho was then added to the stalled EC, which was formed in a similar way as in footprinting experiments using the same DNA template, except that RNA was not radio-labelled, instead P^32^-labelled WT NusA was used. WT NusA has three cys residues (one is in KH1 domain and other two are in AR2 domain). After formation of the stalled EC in the presence of NusA and N, it was washed by phosphate buffer, followed by incubation for 10 min at 37°C with 100 nM of SPDP-labelled WT or Y80C Rho. SPDP-Rho-N cross-linking was not attempted because we observed very high level of non-specific adsorption of N onto the streptavidin beads (Promega), which might had led to spurious cross-linked products outside the EC. Reactions were stopped by non-reducing SDS-sample dye (-βME) and loaded onto a non-reducing 6–10% gradient PAGE, and the products were analysed in a Fuji phosphorimager (Figure 5E and F).

## RESULTS

### Possible modes of N action

We hypothesized that N overcomes Rho-dependent termination by using any one or many of the following mechanisms (Figure 1).

- The *nutR* site (on the right operon) of the lambdoid phages and the *rut* site of the *tR1* Rho-dependent terminator on the nascent RNA overlap with each other (15,24). When the EC is proximal to this site(s), *nut*-bound N-Nus factors-complex may make the *rut* site inaccessible to Rho through a competitive inhibition process (Figure 1A, left panel). A functional competition for the *nutR* site was proposed earlier (25,26). The superimposition of the *tR1* terminator on the *nutR* site provides an unique opportunity to study the molecular basis of the proposed functional competition between a terminator and an anti-terminator. The *nutL* (on the left operon) does not have an overlapping Rho-dependent terminator, and hence we focussed only on the *nutR* site.
- It is possible that instead of competing out Rho from the *nutR* site, N-NusA-Rho forms a ternary complex by co-occupying the same site. In this ternary complex, Rho may get fully inactivated or its conversion into a translocase-competent form becomes slow (Figure 1A, right panel). N only interacts with the tetra-loop region of the *boxB* hairpin (12), and this small footprint may not be enough to occlude Rho from the same site.
- These aforementioned two mechanisms involve N-Nus factor complex mediated inhibition of the Rho action specifically at the *nutR/tR1* site. These inhibitory mechanisms may not be effective when the EC moves farther away from this site enforcing a longer stretch of RNA to be looped out and become accessible for Rho-binding (Figure 1B). To prevent Rho-action in this case(s), it is required to have a termination-resistant configuration of EC by altering the RNA-exit channel formed by N-CTD-RNAP interaction.
- Rho and N uses the same factors, NusA and NusG, for their functions (3,11). It is possible that removal of NusA and NusG from Rho-dependent termination pathway by N makes Rho less efficient (Figure 1C). Sequestration of NusG by N has been speculated earlier (27,28). Functional removal of NusA/NusG from the termination pathway may involve N-mediated remodelling of these host factors.
- N increases the elongation rate of the RNAP (18,29), which enables the EC to overcome pausing signals. Rate of transcription elongation is linked to the efficiency of the Rho-dependent termination through the ‘kinetic coupling’ of the RNAP elongation and the Rho translocation (30). Increase in elongation rate by N may uncouple this kinetic coupling and affect the termination process.

We have tested all these hypotheses in the following sections.

We first investigated the proposed competitive inhibition between N and Rho for the accessibility of nutR/tR1 site, a scenario that is described in Figure 1A (hypothesis A).

### N-NutR interaction is sufficient to overcome Rho near the nutR site

We have used a reporter construct where the *nutR/tR1* sequence is fused to a *lacZYA* cassette (P_lac_-H-19B *nutR/tR1*- *lacZYA*) present as a λRS45 lysogen in a *lac*− strain of MC4100 (GJ5147; Supplementary Figure S1C, single terminator construct; 16). This *nutR/tR1* sequence has the overlapping N (the *nut* site) and the Rho binding sites (the *rut* site), and it is derived from a lambdoid phage H-19B (31). In this construct, the *lacZ* expression occurs only when H-19B N overcomes Rho-mediated termination at the *tR1*, and, on MacKonkey lactose plates, the colonies appear as red or pink. We transformed this GJ5147 strain with the mutagenized library of H-19B N present on the plasmids and screened for white/whitish colonies. We isolated five unique H-19B N mutants defective for anti-termination at *tR1* terminator. All these mutations, R3H, S11F, R15C, R15P and R18P, were located in the *nut-*binding region of N (Supplementary Figure S1A). R15C, R15P and R18P mutations are part of the ARM (..^12^RSRRRER^18^..), which recognizes the *nut* site. Anti-termination assays (see ‘Materials and Methods’ section) revealed that these mutations were severely defective on this single terminator construct (Table 1). Even though the mutagenesis process was random, we obtained point mutations only in the RNA-binding domain of N. Therefore, we hypothesized that the C-terminal RNAP-binding domain of N (CTD) (Supplementary Figure S1A) may not be functionally important for anti-termination on this construct. Dispensability of the c-terminal 14 amino acids of the λN protein was observed earlier (32). To test this, we made several deletions in the CTD of H-19B N. Compared with the point mutants described earlier, the deletions in the last 27 amino acids of H-19B N did not show severe defect (≤1.5-fold compared with the WT) in anti-termination. This defect was partial when the deletions were in the region, 88–95 (Table 1). These results indicated that the CTD of N may not be important for the anti-termination activity on the single terminator construct.

The N CTD interacts with RNAP (13). If CTD–RNAP interaction is not important for this construct, RNAP mutants defective for N binding would be expected to have little effect on anti-termination with this construct. We have used two *rpoC* mutants, R270C and P251S/P254L, which were defective for N-mediated anti-termination on hairpin-dependent terminators (16). Like N CTD mutants, these were also not significantly defective for anti-termination (Table 1, middle column). We also made several deletions in the CTD (73–107 amino acids) of λ N, and tested the same phenomenon using λ *nutR/tR1*construct (Table 1). λ N, like H-19B N, also remained fully active for anti-termination, despite the deletion of major part of its RNAP-binding domain. As N-CTD deletion and RNAP mutants affect the same step in anti-termination, a double mutant would not have additional effects.

Next we tested the *in vitro* anti-termination process using a purified system by carrying out the reactions on an H-19B *nutR/tR1*terminator template (Supplementary Figure S1D, Figure 2A). On this template, WT N anti-terminated very efficiently in the presence of NusA and NusG, whereas the ‘ARM-mutant’, defective for N binding, failed (arginines of ARM,..^12^RSRRRER^18^, are changed to alanine; 16). But, the ΔCTD N (Δ101–127) was able to show significant level of anti-termination (Figure 2B). Similarly, the RNAP mutant, P251S/P254L, also showed partial anti-termination activity on the same template (Figure 2C).

Therefore, we concluded that both under *in vivo* and *in vitro* conditions, the N-RNAP interaction is not essential to overcome the Rho-dependent termination near the *nut* site.

### RNAP modification by N is essential to overcome Rho action away from the nutR site

Next, we explored the mode of inhibition of Rho function by N when the EC moves away from the *nutR* site (Figure 1B; hypothesis C). We created an *in vivo* scenario where the N-modified EC can become a target of Rho when it is away from the *nut* site (as in Figure 1B), by fusing one more Rho-dependent terminator, *trpt'*, (Supplementary Figure S1C, *P_lac_ -nutR/tR1-trpt'-lacZYA*) downstream of the *nutR* site. Similar to the single terminator construct, this one was also inserted into the chromosome as a lysogen (RS1017, Supplementary Table S4). In this construct, Rho-entry site in the second terminator will not face any interference from the N binding, as it is devoid of *nut* site. We repeated the *in vivo* anti-termination assays with WT and different N mutants on this construct. On this template, point mutants in the ARM region remained severely defective for the anti-termination as before, but the CTD deletion mutants, which were largely unaffected on the single terminator construct, were now significantly defective (Table 1, right most columns). Δ121–127 N was also not defective on this template. Probably last seven amino acids of H-19B N are functionally redundant (also see Supplementary Table S1). Similarly, the RNAP mutants also showed defect (Table 1, right most columns). However, this defect was milder compared with the N ΔCTD mutants.

We transcribed the double terminator template (Supplementary Figure S1D, bottom panel; Figure 2D) *in vitro* (Figure 2E) in the presence of different N proteins. Rho terminated in the first terminator region, *tR1*, and the WT N was able to overcome termination both at the *tR1* and *trpt'* terminators. The ARM-mutant N was completely defective. The ΔCTD N showed transcription read through of *nutR/tR1*, but failed do the same through the downstream *trpt'.* Hence, N CTD-RNAP interaction contributes significantly to overcome termination at the *trpt'* terminator. The anti-termination defects of ΔCTD N and RNAP mutants strongly indicated the requirement of the N-RNAP interactions for anti-termination away from the *nut* site, in contrast to the N-mediated inhibition at or near the *nut* site. This essentiality of the N-CTD-RNAP interaction is similar to that observed for hairpin-dependent terminators (Supplementary Table S1; 16,17,19).

The aforementioned results suggest that the N-NTD-*nut* site interaction offers a direct inhibition to Rho, whereas N-CTD-RNAP interaction modifies the EC into a termination resistant form and prevents Rho action through the anti-termination mechanism. Therefore, N uses both inhibition and anti-termination mechanisms to overcome the Rho action.

### N prevents Rho action from the stalled EC

A kinetic coupling between the transcription elongation rate and the translocation rate of Rho determines the efficiency of the Rho-dependent termination (30)*.* We tested whether N-mediated enhancement of transcription elongation rate (18,29,33) plays an important role in overcoming the action of Rho (hypothesis E). We eliminated the effect of N on the elongation rate by stalling the EC on two different immobilized templates using lac repressor as a roadblock (RB complexes; Figure 3A and B). We have earlier reported detailed analyses of Rho-mediated RNA release from the ECs stalled at different sequences and observed that Rho releases RNA very efficiently from those complexes (8, Supplementary Figure S2A). Lac-operator sequence was fused either next to the *nutR/tR1* terminator to stall the EC near the *nut* site (Figure 3A) or after the *tR1-trpt'* double terminator cassette to move the EC further away from the *nut* site (Figure 3B). Rho +1 mM ATP was added to these stalled ECs formed in the presence of WT or different derivatives of N, and the RNA release in the supernatant was measured over a period of time. The RNA release from this RB by Rho was efficient, and the rate of release in the absence of N was ∼1 min^−^^1^ (Figure 3E, Supplementary Figure S2A), and this was comparable with those observed earlier from other RBs (8). We observed the following (Figure 3C–F). Presence of N severely affected the Rho action from the stalled ECs by delaying the RNA release from both the DNA templates. However, Rho eventually overcame the N effect as was evident from the sigmoidal RNA release curves (Figure 3E and F). The ARM mutant N was defective on both the templates, whereas the CTD N was defective only when the EC was stalled further away from the *nut* site. These results showed that (i) the N-*nut* interaction is more important for preventing Rho to act on the stalled ECs near the *nut* site, whereas N-RNAP interaction is equally important for preventing the RNA release from the ECs stalled further away from the *nut* site; and (ii) if sufficient time is allowed, Rho is capable of overcoming the inhibition/anti-termination function of N. This stabilization of EC away from the *nut* site is similar to that observed with the stalled EC at a terminator hairpin (19). Prevention of Rho action by the N-modified stalled ECs also suggested that N can prevent Rho action efficiently without enhancing the elongation speed. However, we cannot rule out that the contribution of the anti-pausing activity of N (33) in overcoming the Rho action because it has been shown that the change in RNAP elongation rate does affect the efficiency of Rho action (30,34).

### N reduces the rate of ATP hydrolysis of Rho at the nut site

The observation that N reduces the rate of RNA release by Rho (Figure 3E and F) and does not fully prevent Rho from acting on the stalled ECs may be owing to the following reasons: (i) N delays the Rho loading onto the *nut* site and also its access to the RNA exit channel of the RNAP; and (ii) N slows down the initiation of ATP hydrolysis and the translocase activity of Rho. We tested the effect of N bound to the *nut* site on the ATPase activity of Rho. We have used the nascent RNA attached to the stalled ECs (as described in Figure 3A and B), either bound to N or in its absence, to activate the ATPase function of Rho. The initial time points of the assay actually measure the ATP hydrolysis activated by the nascent RNA that is still attached to the stalled EC and exhibits the effect of N bound to the EC. The later time points reflect multiple rounds of ATP hydrolysis on the released RNA from the ECs following the Rho-dependent termination. WT N caused a significant delay in initiating the ATP hydrolysis by WT Rho when the EC was near the *nut* site (Figure 4A, compare the rates of ATP hydrolysis; 1.6 nmoles/min/µg Rho versus 0.22 nmoles/min/µg Rho), but the effect of N was modest when the EC was placed away from the *nut* site (Figure 4B; 1.7 nmoles/min/µg Rho versus 1.1 nmoles/min/µg Rho). The ΔCTD N also exerted similar effect as the WT N near the *nut* site, whereas the ARM-mutant N failed to elicit any effect (Supplementary Figure S2B and C). These results indicated that the effect of N on the ATPase activity of Rho is confined near or at the *nut* site.

Further, we hypothesized that this inhibitory effect of N can be overcome by increasing the rate of ATP hydrolysis of Rho. We used a Rho mutant, P235H, with a higher rate of ATP hydrolysis (Supplementary Figure S3A and B; 8). Even in the presence of N, P235H Rho exhibited significantly higher rate of ATP hydrolysis compared with the WT when the EC was near the nut site (Figure 4C, 3.2 nmoles/min/µg Rho versus 1.8 nmoles/min/µg Rho; ≤2-fold). This resulted into a reduction of the N-induced delay of the initiation of ATP hydrolysis. This delay was fully eliminated when the EC was away from the nut site (Figure 4D). Hence, we concluded that the inhibitory mechanism of N at or near the *nut/rut* site involves slowing down of the initiation of the ATP hydrolysis by Rho.

### Co-occupancy of Rho with the N-NusA/G complex at the nut site (hypothesis B)

The reduction of the rate of ATPase activity of Rho by N favours an inhibition model wherein N inactivates Rho at or near the *nut* site, which is likely to be manifested as a functional competition between N and Rho for the same site on the RNA as proposed earlier (26). To achieve the inactivation of Rho, it is possible that the N-NusA/G complex will co-occupy the *nutR/rut* site with Rho. We tested for the co-occupancy by footprinting the *nutR/tR1* site of the nascent RNA of the stalled EC in presence of different factors and also by cross-linking of NusA and Rho both bound to this site.

We stalled the EC in the presence and absence of N by lac repressor bound at a *lacO* site located ∼90 nt away from the *boxB* hairpin of the *nutR/rut* site (Figure 5A, Supplementary Figure S4A). Rho + AMPPNP (a non-hydrolyzable ATP analogue for maintaining the hexameric state) was added to this stalled EC. We have earlier observed that AMPPNP-bound Rho interacts specifically with the *spacer* region of the *nut/rut* site with a small footprint (∼22 nt), which most likely reflects the initial RNA-loading step of Rho (23). This footprint is significantly smaller than that observed during the translocation of Rho (>60 nt) in the presence of ATP hydrolysis. On this stalled EC, N remained functionally active (Supplementary Figure S4B and C).

Footprinting experiments were performed by RNase H and RNase T1-mediated cleavages of the nascent RNA attached to the EC (as in Figure 5A). RNase H cleaves at the RNA:DNA hybrids. We have used DNA oligonucleotides anti-sense to *spacer* and *boxB* regions of the *nutR/rut* site (see next to the gel pictures of Figures 5B and C; Figure 5D). Binding of N, NusA/G and Rho to the *nutR/rut* site will prevent the oligos from binding, which will lead to less sensitivity towards RNase H. RNase H/oligo combination produced cleavages only at RNA:DNA hybrids (Figure 5B lanes without any factors and Supplementary Figure S5). RNase T1 cleaves at the single-stranded G residues. Owing to the presence of two hairpins in the *nutR* site (Figure 5D), only two G residues of the *spacer* and one from the *boxB* were sensitive to T1 (Figure 5C).

Protection from RNase H cleavage was observed in the *spacer* (oligo II-mediated) and *boxB* (oligo III-mediated; Figure 5B) regions when the EC was modified with N+ NusA/NusG. There was no protection in *boxA* and the region downstream of *boxB* (Supplementary Figure S5). Addition of Rho to this N-modified EC did not change the protection pattern. Consistent with our earlier observations (23), Rho on its own produced significant protection of the *spacer* region (Rho only lanes in Figure 5B).

Similar to the RNAse H cleavage pattern, the two Gs of the *spacer* region were protected by Rho from the RNAse T1 cleavage, whereas all the three Gs (the third G is from the *boxB* loop) were protected when N +NusA/NusG were present (Figure 5C; protected part is indicated by arrows above the intensity profile). Presence of Rho did not change this protection pattern. Protection of *spacer* and *boxB* by NusA and N is consistent with the fact that NusA binds to *spacer* and N to the tetra-loop of the *boxB* (12,14). Rho and NusA bind to the same *spacer* region. The similar footprinting pattern of the N-Nus complex both in the absence and presence of Rho suggests that either Rho was not associated to the same site or it co-occupies the site with N-Nus complex without changing the nature of the protection. However, delay in the rate of Rho-induced RNA release (Figure 3) or ATP hydrolysis (Figure 4) from the N/NusA-modified stalled EC may favour the proposal of co-occupancy of Rho with the N-NusA complex at or near the *nut/rut* site.

To establish the co-occupancy of N, NusA and Rho at the *nut* site more convincingly, we monitored the cross-linking efficiency of Rho and NusA at the *nutR/rut* site of the nascent RNA of the RB complex (as in Figure 5A). We have probed NusA-Rho cross-linking because both of them have overlapping binding sites at the *spacer* region and therefore, if they co-occupy the *nutR/rut* site, chances of cross-linking between them will be higher. Also stable binding of NusA to the *spacer* requires presence of N at the *boxB* hairpin. Hence, NusA-Rho cross-linking effectively provides the evidence for the N-NusA-Rho co-occupancy. We have used a bi-functional cross-linker LC-SPDP (with a linker length of ∼15 Å; Supplementary Figure S6), which specifically forms inter-molecular cross-links between primary amines (e.g. lysines) of a cysteine-less protein, C202A Rho (the only cysteine of Rho is changed to an alanine; 22) and the cysteine side-chains of NusA (having three cysteines). The amine-cysteine Rho-NusA cross-linked product can be identified on a non-reducing SDS-PAGE using radiolabelled NusA and by comparing with appropriate molecular weight markers.

For the cross-linking experiments, similar RB complexes as described in Figure 5A were formed in the presence of N and NusA. SPDP-labelled WT or Y80C Rho was added to the N-NusA modified RBs in the presence of 1 mM AMPPNP. We observed two cross-linked products, Rho-NusA (monomers of both NusA and Rho) and 2Rho-NusA (2 Rho sub-units and monomer of NusA) (Figure 5E, left panel), only in the presence of SPDP-labelled WT Rho. 2Rho-NusA species might have formed by cross-linking of two SPDB molecules from two subunits of Rho and two cys residues of the same NusA molecule. These products were not seen either in the presence of unlabelled WT Rho or SPDP-labelled Y80C Rho (21). Latter is an RNA-binding defective mutant of Rho and never showed any association with the EC (23). These products were also not observed in the absence of transcription EC (Figure 5F, right panel). We concluded that Rho can specifically be cross-linked to NusA present in the N-NusA complex bound to the *nut/rut* site of the EC, which is stalled 90 nt downstream of the *boxB* hairpin. This result strongly supports the proposal of co-occupancy of Rho-N-NusA and the possibility of Rho-NusA interaction at the *nut/rut* site.

We further measured the co-occupancy of Rho with N-NusA at the *nut/rut* site by using a direct binding assay of the former to the RB described in Figure 5A. We added a radio-labelled WT Rho to the stalled EC formed on an immobilized DNA template. Fraction of Rho obtained in the pellet fraction was the measure of association with the EC (Supplementary Figure S7A; 23). Presence of N and Nus factors with the stalled EC did not show any effect either on the amount of Rho-binding or on its binding kinetics (Supplementary Figure S7B). This association of Rho was through the *nut/rut* site of the nascent RNA because a Rho mutant, Y80C (21), defective for RNA binding, did not show any association to EC (Supplementary Figure S7C; 23). These results further support the proposal for N-NusA-Rho co-occupancy at the *nut/rut* site.

Finally, we probed the functional consequences of the co-occupancy of N and Rho on the *nutR/rut* site (Supplementary Figure S7D). We used the template described in Figure 5A. On this template, we can measure the Rho-dependent termination of the stalled EC at the *lac* operator site and the anti-termination activity of N at the hairpin-dependent terminator *tR'*. We, at first, made stalled ECs in the presence of lac repressor (Supplementary Figure S7D; RB, lanes 1 and 8), and they were capable of elongation when IPTG (isopropyl β-D-1-thiogalactopyranoside) was added in the absence of Rho [lanes 2 and 9, *tR'* and run-off (RO) products]. The N-modified stalled EC was also observed to read through the *tR'* terminator efficiently to yield the RO product (lane 9). In the absence of N, Rho terminated the stalled EC, which was evident from the accumulation of the RB product over time (lanes 3 to 7; Supplementary Figure S7E for the plot). In the presence of N, not only anti-terminated products (RO) were formed but also a slow accumulation of RB at the lac operator site was observed (lanes 10 to 14; compare the −N and +N plots in Supplementary Figure S7E). These results suggested that Rho can still terminate in the presence of N albeit with a slower rate, which further confirmed the co-occupancy of N, NusA/G and Rho factors at the *nutR/rut* site.

### Suppressor mutations in Rho

**Figure 6.**
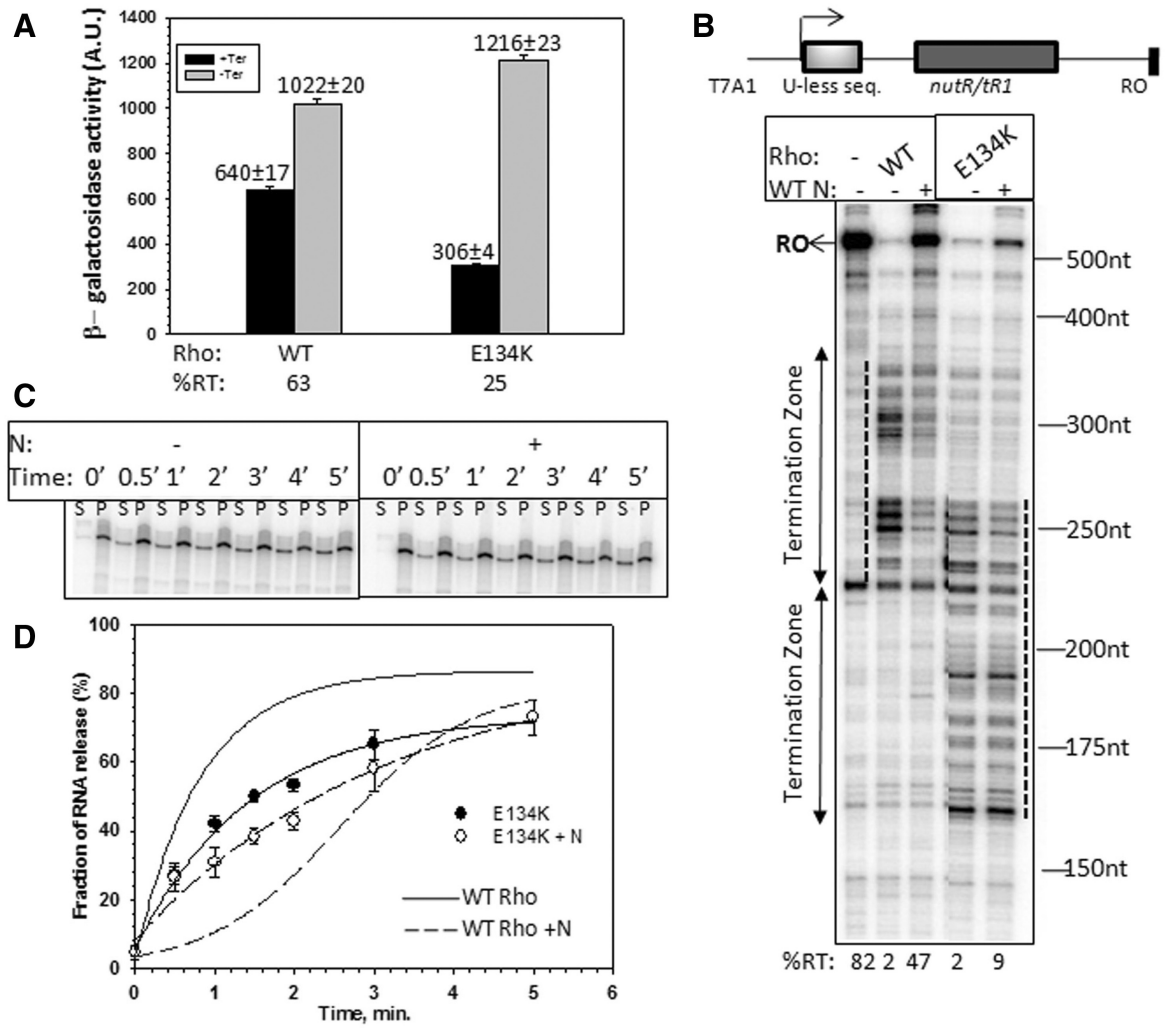
Suppression of N by Rho mutants. (**A**) *In vivo* anti-termination activity of H-19B N in presence of WT and Rho mutants. H-19B *nutR/tR1-trpt'* terminator fused to a *LacZYA* reporter was used for this purpose. The average β−galactosidase activities are indicated above the bars. The calculations of anti-termination activity (%RT) and the assays were performed in a similar way as described in Table 1. (**B**) Autoradiogram showing the *in vitro* transcription assays at *H-19B nutR/tR1* terminator under indicated conditions. Termination zones in presence of WT and mutant Rho are indicated by two headed arrows and also with dotted lines. Size markers are indicated. Template used for the assay is shown above the gel. Amounts of RO transcript in each lane are indicated below the gel. The concentrations of Rho (WT / mutant) and H-19B N were 50 nM and 25 nM, respectively. The assays were done at 25 µM NTPs and in the presence of 200 nM NusG and 300 nM NusA. (**C**) Autoradiogram showing the amount of RNA released by E134K Rho, both in the absence or presence of WT H-19B N from the stalled EC formed on the T7A1-*nutR/tR1-lacO* template similar to that described in Figure 3A. Concentrations of E134K Rho and H-19B N were 50 nM and 100 nM, respectively. ‘S’ denotes half of the supernatant, and ‘P’ denotes the rest of the sample. RNA release was estimated as, [2S]/([S] + [P]) and plotted against time (**D**) both in the absence or presence of WT H-19B N. RNA release by WT Rho on the same template (taken from Figure 2A) is indicated by solid and dashed curves only. In all, 300 nM NusA and 200 nM nusG were present in all the cases.

To identify whether any other steps in Rho-dependent termination is affected by N, we looked for suppressor mutations in Rho, which enable it to overcome N. We randomly mutagenized the *rho* gene and screened for mutants, which retained the termination function even in the presence of WT N (see Supplementary Methods). We isolated a Rho mutant, E134K. Another Rho mutant, P103L, was reported to prevent growth of a certain λ phage, λr32, probably by overcoming the N function (28,35,36). We measured the anti-termination efficiency of N at the *nutR/tR1-trpt'* double terminator construct fused to the *lac-ZYA* reporter, in the presence of E134K Rho in a similar way as performed in Table 1 (see Materials and Methods). The anti-termination efficiency of N reduced significantly in the presence of this Rho mutant (Figure 6A; compare %RT values). It was also defective in supporting the growth of both the H-19B and λ phages (Supplementary Figure S8).

Next, we tested the *in vitro* anti-termination activities of H-19 B N in the presence of the E134K Rho using the H-19B *nutR/tR1* terminator template (Figure 6B). Amount of RO product, the measure of *in vitro* anti-termination by N, was reduced by 5-fold compared with WT when E134K was present (compare the %RT values shown at the bottom of Figure 6B). Also E134K induced early termination.

We then tested whether E134K Rho can overcome N-mediated delay in RNA release from a stalled EC. We first formed a stalled EC modified with N and NusA (similar to that in Figure 3A). Interestingly, unlike its effect on WT Rho, WT N was unable to prevent RNA release by E134K even from the stalled EC (Figure 6C and D; compare the release kinetics with WT Rho).

Above results strongly indicated that E134K Rho functions as a suppressor of the N.

### Unusual dependence of E134K Rho on NusA

Next, we explored the mechanism of suppression of N function by E134K Rho. We hypothesized that E134K Rho might have gained unusual transcription termination properties, which helped it to overcome the anti-termination by N. The slow rate of RNA release by E134K Rho from a stalled EC (Figure 6D) suggests that it may have termination defect, and its early termination behaviour in the presence of NusA/NusG (Figure 6B) indicates its dependence on these factors. Hence, we probed the termination properties of this Rho mutant in more details.

**Figure 7.**
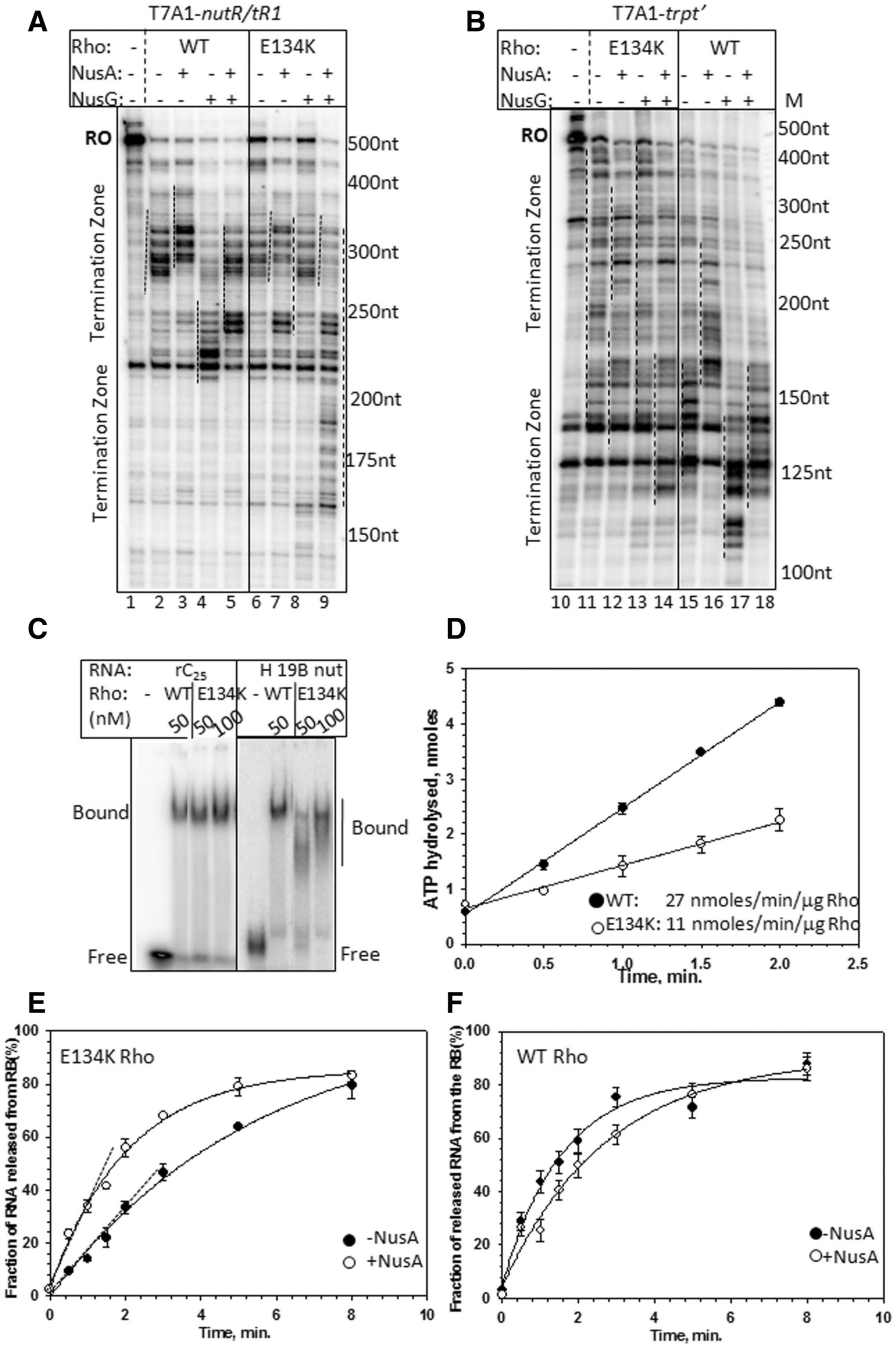
NusA-dependence of E134K Rho*.* Autoradiograms showing the steady state single round *in vitro* transcription termination in the presence of WT and E134K Rho on H-19B *nutR/tR1* terminator (**A**) and on *trpt'* terminator template (**B**). Termination region is indicated by dotted lines next to the transcript bands. RO denotes the RO product. The concentration of Rho, NusA and NusG were 50 nM, 300 nM and 200 nM, respectively. The assay was carried out at 25 µM NTPs. (**C**) Autoradiogram of a native PAGE showing the migrations of the free and Rho-bound rC_25_ oligo and H-19B *cro* RNA. rC_25_ is a 25 mer poly(C) RNA, and H-19B *cro* RNA contains the *tR1* terminator sequence from the lambdoid phage H-19B. Free and bound fractions of the RNA are indicated. Binding events were performed in the presence of ATP analogue, AMPPNP. In all the experiments, RNAs were labelled with P^32^. The concentration of Rho is indicated. Concentration of labelled oligo was 10 nM. (**D**) ATPase assay of Rho in the presence of poly(C) as RNA cofactor. Representative plots showing the amounts of [γ-^32^P]ATP hydrolysed with time. The data were fitted by linear regressions using SIGMAPLOT. Rates of ATP hydrolysis are indicated as nmol/min/µg of Rho. Fractions of RNA released by E134K (**E**) and WT Rho (**F**) are plotted against time both in the absence or presence of NusA from the stalled EC inside H-19B *nutR/tR1* termination region. The DNA template was same as in Figure 3A.

At first, we measured the *in vivo* termination efficiency of E134K Rho using the single and double terminator cassettes (same as in Table 1), and observed severe termination defects, especially on the single-terminator construct (Supplementary Table S2). Next, we investigated the *in vitro* termination efficiency of E134K Rho both in the presence and absence of NusA and NusG. We used two separate templates, *nutR/tR1* or *trpt'* terminators fused to the T7A1 promoter (Figure 7A and B)*.* In case of WT Rho, presence of NusA delays the termination window, whereas NusG induces early termination and an intermediate effect is observed in the presence of both these factors (lanes 2–5 of 7A and 15–18 of 7B; 37). In the absence of any factor, E134K Rho showed termination defect on both the terminators (increase in the amount of RO; lane 6 of 7A and lane 11 of 7B). Unlike WT Rho, NusG on its own was unable to induce early termination or improve the efficiency of E134K Rho (lane 8 of 7A and lane 13 of 7B). Interestingly, instead of delaying the termination, NusA improved the termination efficiency and together with NusG, made E134K Rho extremely efficient and early terminating (lane 7 and 9 of 7A; lanes 12 and 14 of 7B).

It is possible that E134K does not bind to NusG and has acquired an unusual property of NusA-binding; therefore, we tested the binding of E134K Rho to NusA and NusG by pull-down assays. We did not observe any E134K–NusA interaction or defect in E134K–NusG complex formation (Supplementary Figure S9A and B). NusG–Rho interaction stimulates the termination process by increasing the speed of RNA release and does not affect either RNA-binding or RNA-dependent ATPase activities of Rho (21). It is possible that E134K may have defect in these early steps of termination, and NusA helps to overcome these defects by a direct interaction at the *nutR*/*rut site* (Figure 5E).

Therefore, we, at first, tested the RNA-binding and RNA-activated ATPase activity of E134K Rho. We assessed the RNA binding of E134K by gel-shift assays, using a short RNA, rC_25_, and a natural RNA from H-19B phage having the *nut/rut* site (38). Compared with the WT Rho, E134K mutant showed similar affinity for the shorter RNA, but significantly weaker RNA binding (appearance of a smear is an evidence for weaker association) for the longer RNA, which passes through the secondary RNA binding sites (H-19B RNA; Figure 7C). It also demonstrated slower rate of ATP hydrolysis even with the strong Rho-substrate polyC (Figure 7D). These results suggest that E134K Rho is defective in secondary RNA binding, which is consistent with its location near the path of the RNA in the central hole of the Rho structure (Supplementary Figure S10).

The defects described earlier may give rise to a slow rate of RNA release by E134K from a stalled EC, and NusA may improve this rate. We measured the RNA release by E134K from a stalled EC, similar to the one described in Figure 3A and checked the effect of NusA on it. NusA improved the rate of RNA release of E134K significantly (Figures 7E; compare the slopes), which was in contrast to what was observed for WT Rho (Figure 7F). Hence, NusA improves the termination efficiency of E134K by increasing its rate of RNA release, and this might have stimulated NusG function indirectly (Figure 7A and B, lanes 9 and 18). The RNA-binding function of NusA is observed only when it is a part of the EC. Hence, we did not attempt to follow the effect of NusA on E134K on either the RNA-binding or RNA-dependent ATPase assays outside the EC.

Presence of NusA very close to Rho at the *nut/rut* site (Figure 5E) can help E134K mutant to properly bind the RNA in its secondary binding site(s) and to speed-up its isomerization steps leading to a translocase competent state. This could explain the unusual functional dependence of this Rho mutant on NusA.

This dependence of E134K Rho on NusA might have perturbed the N-NusA interaction at the nut site, which in turn affected the anti-termination function of N. And this could be the likely mechanism for the suppressor action of E134K.

## DISCUSSION

### A multi-pronged strategy of N to overcome Rho

The small anti-terminator protein N has three interacting regions (Supplementary Figure S1A). They interact with the *nut* site on mRNA (12), with the *nut-*bound NusA (13) and with the RNAP (13). Here, we show that N uses all these three interaction modules to use a multi-pronged strategy to overcome the Rho action.

- N-NusA-Rho forms a ternary complex at the *nut/rut* site (Figure 5), and this configuration inactivates Rho (Figure 4), which in turn slows down the translocation and the RNA release kinetics of Rho (Figure 3). Most likely, the presence of N and NusA at the *nut* site affects the proper placement of the downstream RNA into the central hole of the Rho hexamer (mechanism 1 in Figure 1A). This inhibition of Rho function at the *nut* site does not require N induced modification of the EC (Table 1).
- N-CTD interacts with RNAP near the RNA exit channel and may use this channel to penetrate a part of its ‘thread-like’ CTD into the interior of the EC (16,17). This interaction becomes important to prevent Rho action when the EC moves away from the *nut* site allowing Rho to freely bind and translocate along the nascent RNA present between the *nut* site and the EC (Table 1, Figure 1B). Although it has not been proved, the RNA exit channel could be the likely area through which Rho gains access to the RNAP. Presence of N-CTD and NusA-NTD (11) in the vicinity of the exit channel may function as a lid to the Rho-access point and prevent/delay the putative Rho-RNAP interaction (mechanism II, Figure 1B).
- The unusual dependence of the E134K Rho on NusA for efficient termination (Figure 7) and its suppression activity of N function (Figure 6) led us to propose that Rho and N compete for the same NusA molecule bound to the *nut* site. N-NusA interaction removes NusA from the Rho-dependent termination pathway and makes the latter process less efficient on the tR1-like terminators (mechanism III, Figure 1C).
- NusG stimulates N activity *in vitro* (29) and was shown to be a part of the *in vivo* anti-termination process (39). On the other hand, Rho interacts with the C-terminal domain of NusG (22,40), and this interaction is essential for an efficient termination. We observed that *in vivo*, NusG-CTD mutants defective for Rho binding (22) did not have any effect on N function (Supplementary Table S3). Hence, we concluded that N functions independent of Rho-NusG CTD interaction. However, incorporation of NusG into the N-anti-termination machinery can alter NusG-NTD-β' clamp helices interactions, which in turn may perturb the Rho-NusG complex formation.

### NusA-remodelling, as a possible anti-termination mechanism of the anti-terminator, N

NusA interacts with RNAP and also with the nascent RNA emerging out of the EC (41,42). It is an important component for both the termination and the anti-termination processes. NusA improves the efficiency of hairpin-dependent termination likely by stabilizing the RNA hairpins of the terminators (42,43,44). It is also involved in Rho-dependent termination (37,42,45). On the other hand, anti-terminators like N- and Q-functions are highly NusA-dependent (11,42). N makes NusA more specific to *nut* site (14) and also changes its mode of interaction with RNAP (19), whereas in the presence of Q protein, NusA forms a shield at the RNA exit channel (46). Specific interaction of N with NusA leads to a ‘NusA-remodelling’, and its subsequent removal from the termination pathway may be by following means.

- As Rho and NusA binding sites (‘*spacer*’ region; also Figure 5) at the *nutR/rut* overlap, the high affinity N-NusA interaction at the *nut* site may make NusA unavailable to Rho during its loading to and activation by the *nut* RNA.
- NusA-β-flap interaction near the RNA exit channel (11) could be instrumental in helping Rho to access the interior of the EC. The proposed N-induced (19) changes in the NusA-RNAP interaction is likely to affect the putative Rho-RNAP interaction or the terminator hairpin folding at the RNA exit channel.

We propose that ‘NusA-remodelling’ could be an important mechanism used by N to overcome both the Rho-dependent and -independent terminations in addition to stabilizing the transcription ECs.

### What is the role of NusA in the Rho-dependent termination?

Involvement of NusA in Rho-dependent termination has been implicated in different reports (37,45,47,48). The role of NusA in this process is still unknown. Here, for the first time, we report a Rho mutant, E134K, whose function is highly dependent on NusA and not on NusG (Figure 7)*.* NusA improves the termination efficiency of E134K by increasing the rate of RNA release and stimulating the NusG function. We suggest that the secondary RNA binding defect of E134K (Figure 7C and D) is rectified by NusA-mediated chaperoning of the RNA into the secondary channel, and this stabilization of RNA in the central hole may also stimulate the NusG function. Based on these results, we propose that the role of NusA is important for a subset of Rho-dependent terminators, where Rho-loading onto the RNA and subsequent activation step(s) are rate limiting. In these terminators, owing to the structural constraints, the nascent RNA cannot be placed properly into the central hole of the hexameric Rho, thereby affecting its ‘open’ to ‘close’ isomerization step(s) and the rate of initiation of ATP hydrolysis. Analogous to the chaperoning role of NusA for the Rho-independent terminators with imperfect RNA hairpins, we envisioned that it also functions as a RNA-chaperone to guide the nascent RNA into the central hole of the hexameric Rho.

### Spatial relationship among N, NusA, NusG and Rho on the RNAP

Aforementioned discussion indicates a spatial relationship among the factors of the termination and anti-termination machinery on the EC, which probably enables both N and Rho to compete for the same NusA and NusG molecules bound to the RNAP surface and use them as vehicles to access the interior of RNAP. On interacting with these two factors, Rho and N are likely to alter the conformations of the β-flap domain near the RNA exit channel and the β'-clamp helices near the non-template strand at the active centre. Rho-NusG CTD interaction not only helps Rho to be recruited to the EC but may also alter the NusG-NTD -β' clamp helices interactions. On the other hand, N is likely to change conformations in the RNA exit channel by directly interacting with NusA and bringing about alterations in the flap-domain. Therefore, it is likely that these two structural elements of RNAP on their own and cross-talk between them play pivotal roles in both termination and anti-termination processes.

## SUPPLEMENTARY DATA

Supplementary Data are available at NAR Online: Supplementary Tables 1–3 and Supplementary Figures 1–10.

## FUNDING

Department of Biotechnology (DBT); Government of India and intramural funding of Centre for DNA Fingerprinting and Diagnostics (CDFD); Swarnajayanti fellow of Department of Science and Technology, Government of India (to R.S.); senior research fellowship for DBT (to G.M.); post-doctoral fellowship (to D.D.); Indian Council of Medical Research (ICMR) senior research fellowship (to S.M.). Funding for open access charge: Waived by Oxford University Press.

*Conflict of interest statement*. None declared.

## Supplementary Material

Supplementary Data
