# Supplementary material for: A multipronged strategy of an anti-terminator protein to overcome Rho-dependent transcription termination
Source: Nucleic Acids Res. 2012 Sep 29;40(22):11213–28. doi: 10.1093/nar/gks872 (PMC3526286; doi:10.1093/nar/gks872)
Supplement: Supplementary Data [file supp_40_22_11213__index.html]

A multipronged strategy of an anti-terminator protein to overcome Rho-dependent transcription termination — A multipronged strategy of an anti-terminator protein to overcome Rho-dependent transcription termination — Supplementary Data 

# A multipronged strategy of an anti-terminator protein to overcome Rho-dependent transcription termination

## Supplementary Data

files

**Files in this Data Supplement:**

- Supplementary Data - pdf file
