# Supplementary material for: A multipronged strategy of an anti-terminator protein to overcome Rho-dependent transcription termination
Source: Nucleic Acids Res. 2012 Sep 29;40(22):11213–28. doi: 10.1093/nar/gks872 (PMC3526286; doi:10.1093/nar/gks872)
Supplement: Supplementary Data [file supp_gks872_nar-01635-r-2012-File009.pdf]

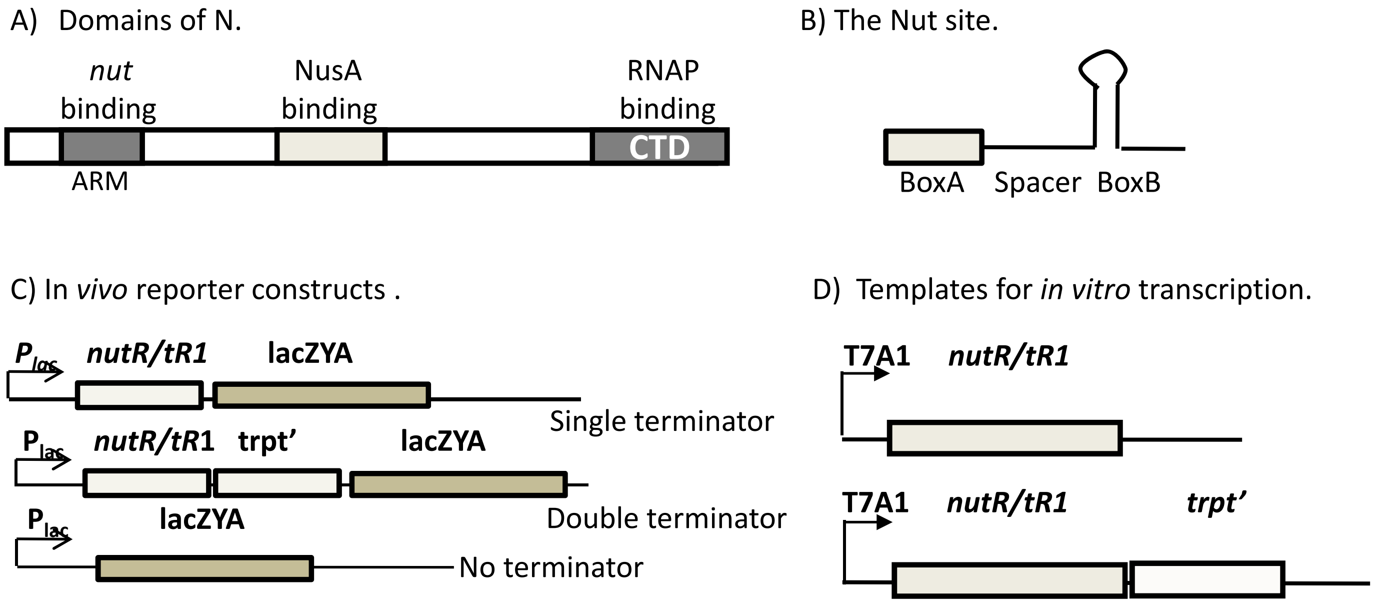

Figure S1. Descriptions of N, Nut site and different constructs. A) Different functionally important regions of N protein. N interacts with *boxB* hairpin, with the Arginine Rich Motif (ARM) in its N-terminal and with RNA Polymerase with its C-terminal. The central portion binds to NusA. B) Organization of nut site. N binds to the loop region of the hairpin. NusA binds to the spacer region. Other Nus factors assembles on *boxA* sequence. C) Different reporter cassettes used for measuring the *in vivo* antitermination by N in the by β-galactosidase assays. Single terminator has a *nutR/tR1* rho dependent terminator. In the double terminator constructs, *nutR/tR1* is followed by another Rho dependent terminator, *trpt'*. In the 'No terminator' construct, the promoter P_{lac} is immediately followed by *lacZYA* without any Rho dependent terminator. The ratios of b-galactosidase activity in the presence and absence of the terminators gave the measure of antitermination. D) Templates used for *in vitro* transcription assays. Transcription is initiated from a strong T7A1 promoter .

A)

[NTP]	RB	Rate (min^{-1})	Reference
1 mM ATP	at +161 of trpt' (Catalytically competent RB)	1.4	(Dutta *et al.*, 2008)
1 mM ATP	In H-19B TR1 termination zone	0.9	(Kalarickal *et al.*, 2010)
1 mM ATP	In H-19B TR1 termination zone	1.1	This study

B)

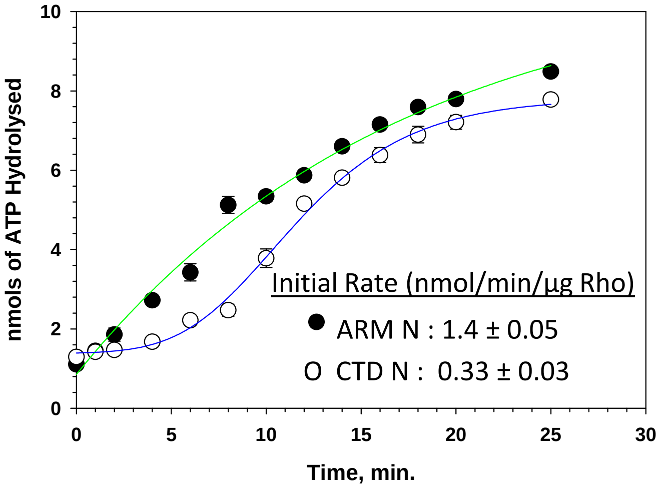

C)

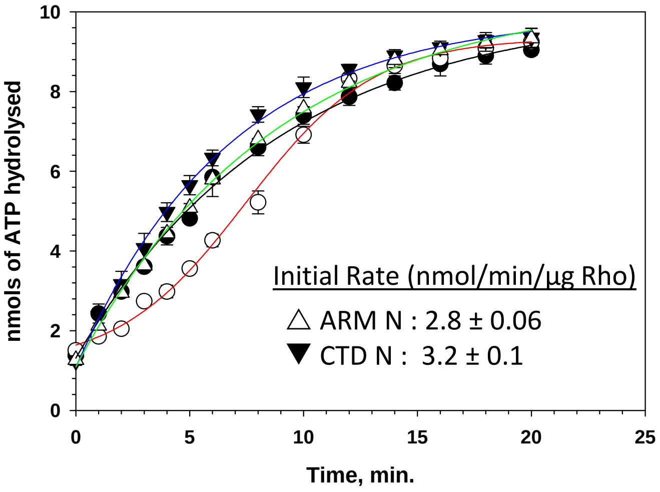

Figure S2: A) Rates of RNA release from stalled ECs (RB) on different terminators by Rho. **B) and C) Effect of N derivative on the ATPase activity of Rho from stalled elongation complexes at different distances from *boxB*. A**) Stalled EC near the *nut* site and **B**) farther away from the *nu*t site. Same templates described in figure 2 are also used here. Fraction of released of inorganic phosphate (Pi) from [γ -^{32}P]ATP by Rho is plotted against time in the absence or presence of WT H-19B N, CTD N or ARM N as indicated. Concentrations of Rho and H-19B N were 100 nM each. 300 nM NusA and 200 nM NusG is present in all the cases. Error bars were calculated from three measurements.

A)

P235H at *tR1*

Rho	[NTP] mM	Rate of ATP Hydrolysis nmol/min/μg Rho	Fold increase
WT	1.0	1.6 ± 0.1	(1)
P235H	1.0	3.2 ± 0.07	2

B)

P235H at *tR1- trpt'*

Rho	[NTP] mM	Rate of ATP Hydrolysis nmol/min/μg Rho	Fold increase
WT	1.0	1.7 ± 0.2	(1)
P235H	1.0	6.2 ± 0.6	3.7

Figure S3: The rate of ATPase activity of WT and P235H Rho from stalled EC at different distances from *nut site*. Rate of ATP hydrolysis is compared between WT and P235H Rho during ATPase assay on the ECs stalled at a single terminator *tR1* **(A)** and after the double terminator construct **(B**) similar to that described in figure 3 .

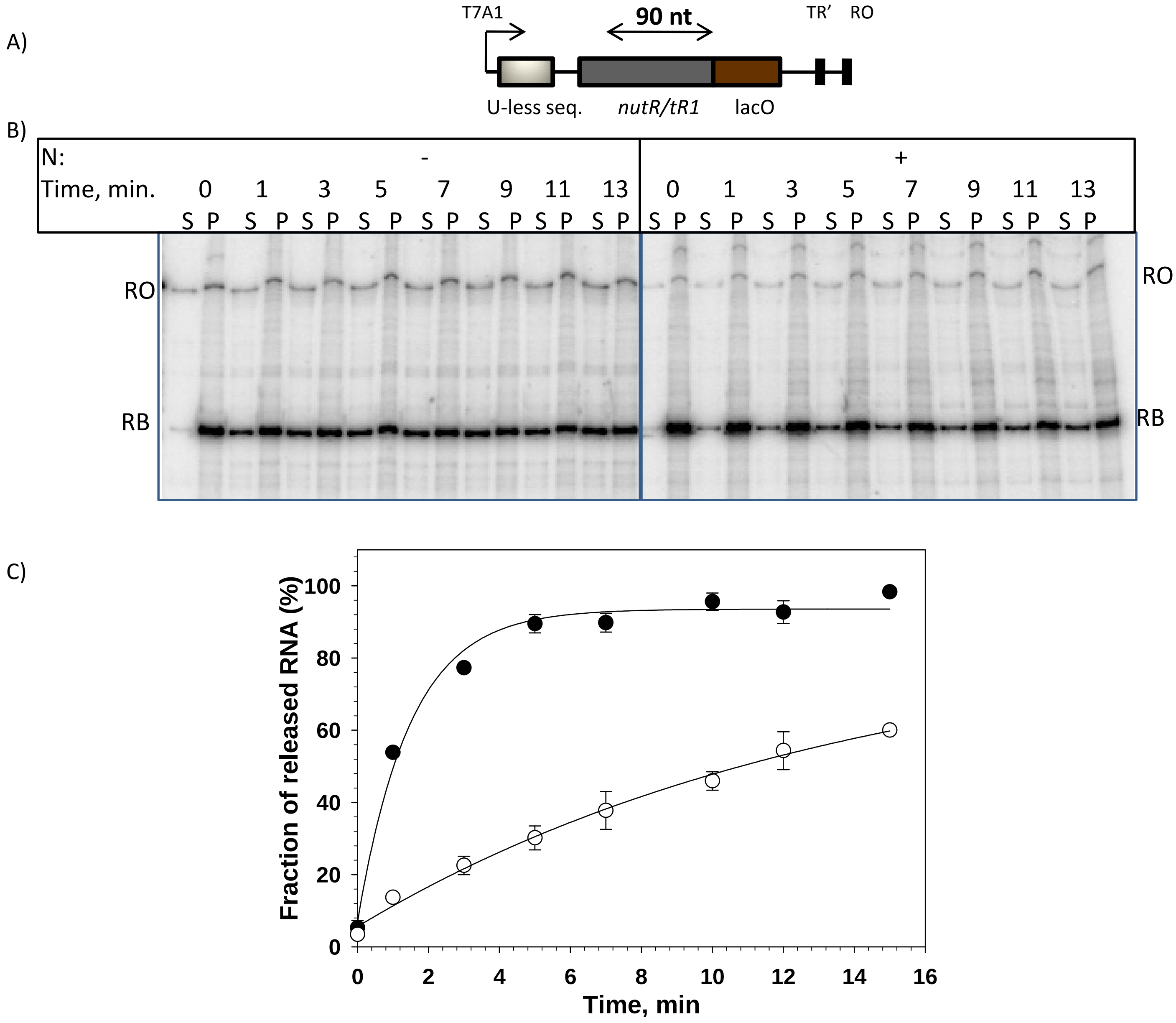

Figure S4: Effect of N on the Rho-mediated RNA release from stalled EC formed very close to *nutR boxB*. A) Cartoon showing the design for making stalled EC inside the H-19B *nutR*/*tR1* terminator region using a *lac* repressor as a road-block. In this design, EC will be ~90nt from the *boxB* hairpin. **B)** Autoradiogram showing the amounts of RNA release by Rho from the stalled EC, both in the absence or presence of WT H-19B N. Concentrations of Rho and H-19B N were 50 nM and 100 nM, respectively. ‘*S*’ denotes half of the supernatant, and ‘*P’* denotes the rest of the sample. **(C)** Fraction of RNA release was estimated from [2*S]/([S]+[P])* and was plotted against time both in the absence or presence of WT H-19B N. In all the experiments 300 nM NusA and 200 nM NusG were present. Slow release of RNA indicates that N remained functionally active on this construct.

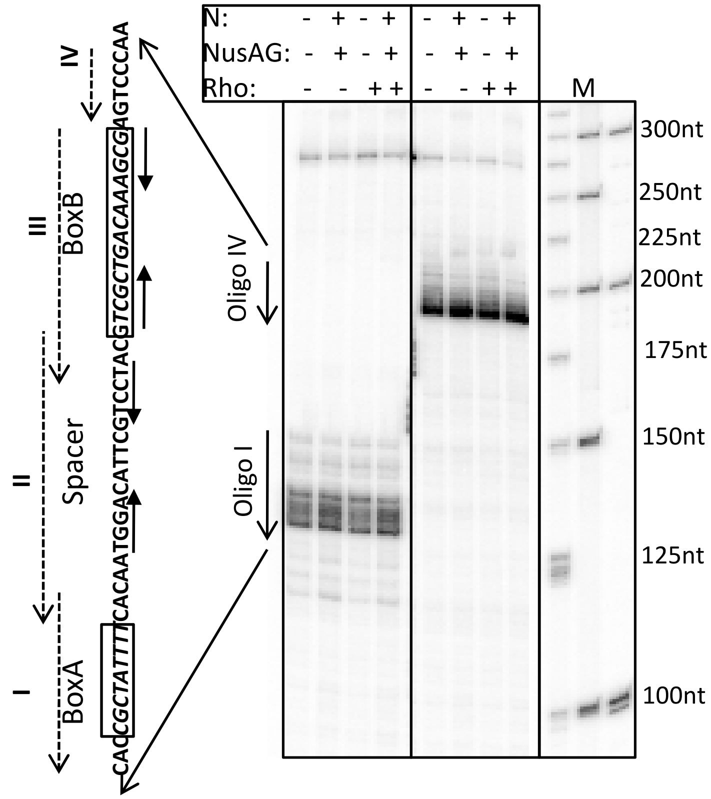

Figure S5: RNAseH footprinting of the nascent RNA of the stalled elongation complex as described in figure 5. RNaseH cleavages were obtained using oligo I (RS662) in the boxA region and oligo IV (RS665) located just beyond the boxB region. Experiments were done in the same way as in figure 5. These two regions did not show any N or Rho mediated protection. The nucleotide size markers and the *nut/rut* regions are indicated.

LC-SPDP (Sulfosuccinimidyl 6-[3’(2-pyridyldithio)-propionamido] hexanoate

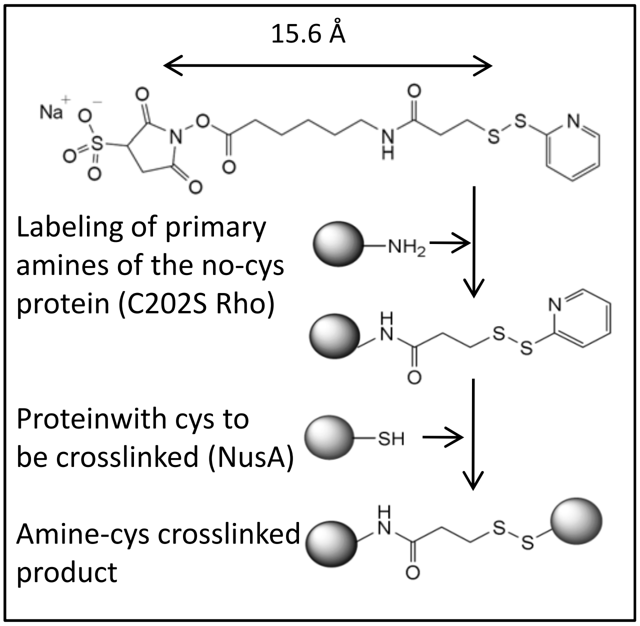

Figure S6: Cartoon showing the chemical structure of the cross-linker LC-SPDP with a spacer length of 15.6 Å between the cross-linking groups.The reaction pathway for amine-cysteine cross-linking between two protein molecules is also shown. The amine groups of a zero-cys derivative (C202S) of Rho were derivatized with SPDP that was subsequently cross-linked to cys containing NusA.

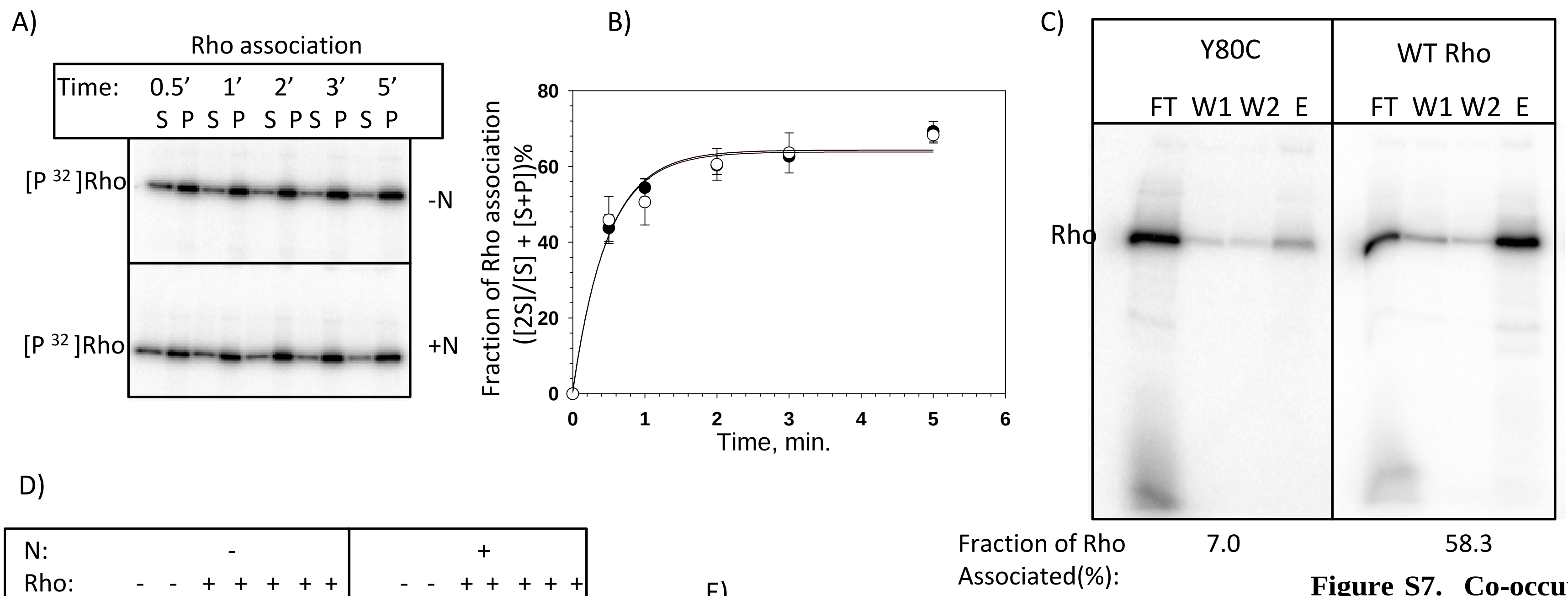

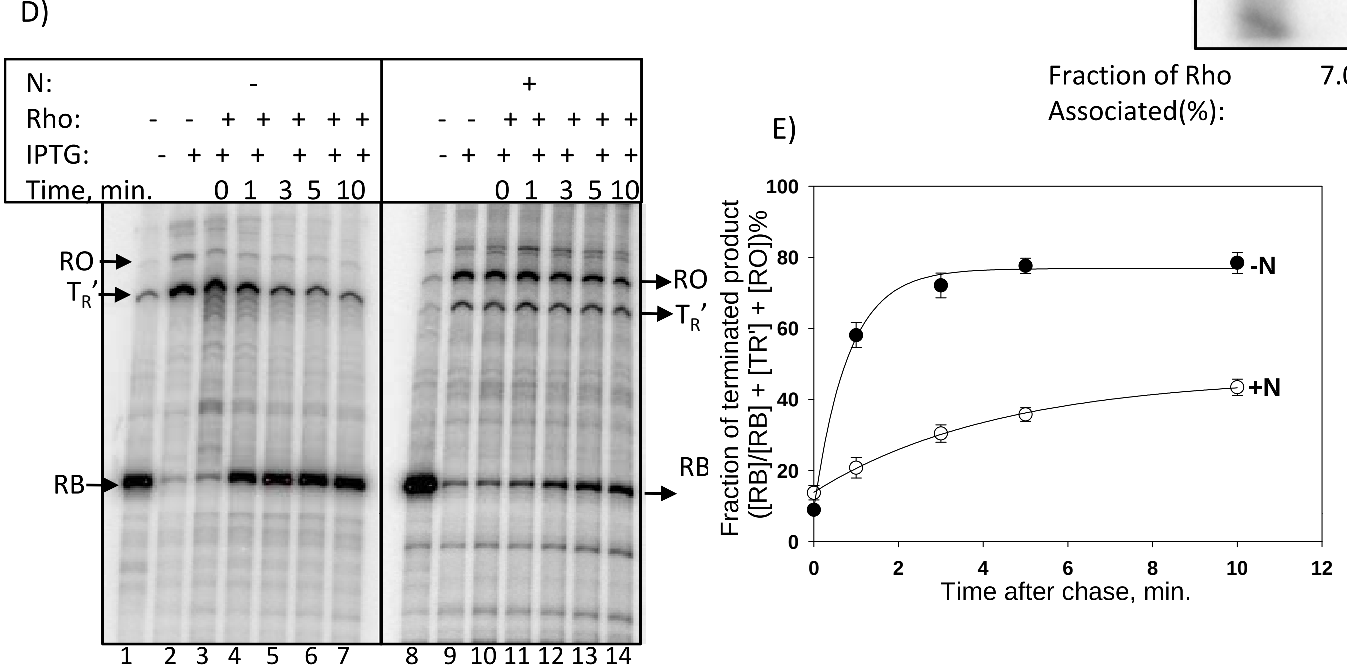

Figure S7. Co-occupancy and functional tests for N-NusA-Rho co-occupancy at the *nut* site. **A)** Auto-radiograms of the radio-labelled WT Rho showing its association with EC (same as described in Figure 5E and F) at different time points in the presence and absence of N. 25 nM Rho and 100 nM WT H-19B N were used. 300 nM NusA and 200 nM NusG were also present in the reaction. Rho was complexed with 1 mM AMPNP. **B)** Fraction of Rho association is plotted against time both in the absence and presence of WT H-19B N. Error bars were calculated from two independent measurements. **C)** Association of WT and Y80C Rho with the stalled EC**.** Amounts of radio-labeled Rho associated with different fractions. Pellet fractions (P) indicate the amount associated with the stalled EC. Experimental procedures has the details of this association study. Rho was complexed with the ATP analogue AMPPNP to avoid its non-specific adsorption on the magnetic beads (23) and also to maintain its hexameric state. **D**) Autoradiogram showing *in vitro* chasing of the Rho bound stalled EC in presence/absence of N. The RB on the template described in A, was formed either in the absence (left panel) or presence (right panel) of 100 nM WT N. These were incubated with 50 nM Rho + 1mM ATP and were chased with 1 mM IPTG + 0.25mM NTPs at indicated time points. Terminated/un-chased products at the lac operator site are denoted as RB. tR' and RO indicate the terminated product at tR' terminator and run-off product, respectively. 300 nM NusA, 200 nM NusG were also present in the reactions. **E)** Fractions of Rho mediated terminated product (RB) are plotted against time both in the absence or presence of WT H-19B N. Error bars were calculated from two independent measurements.

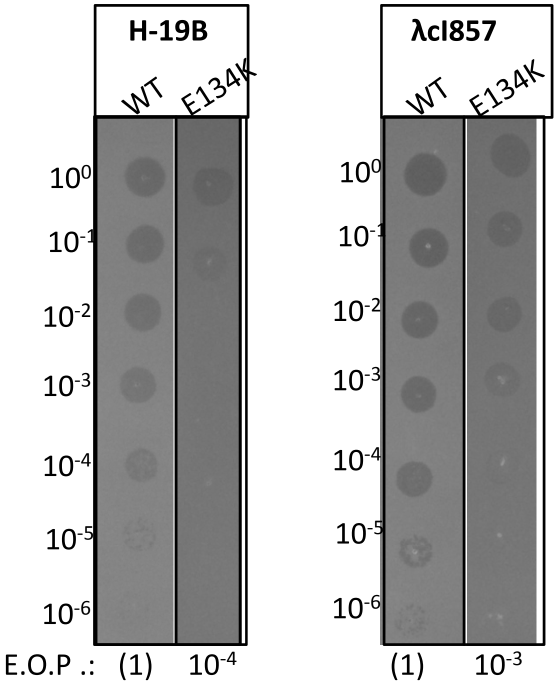

Figure S8. *In vivo* phage growth assay for H-19B and λcI857 phages, in the presence of WT and Rho mutant. Dilutions of phages are indicated beside the picture and E.O.P. (efficiency of platting) are indicated below.

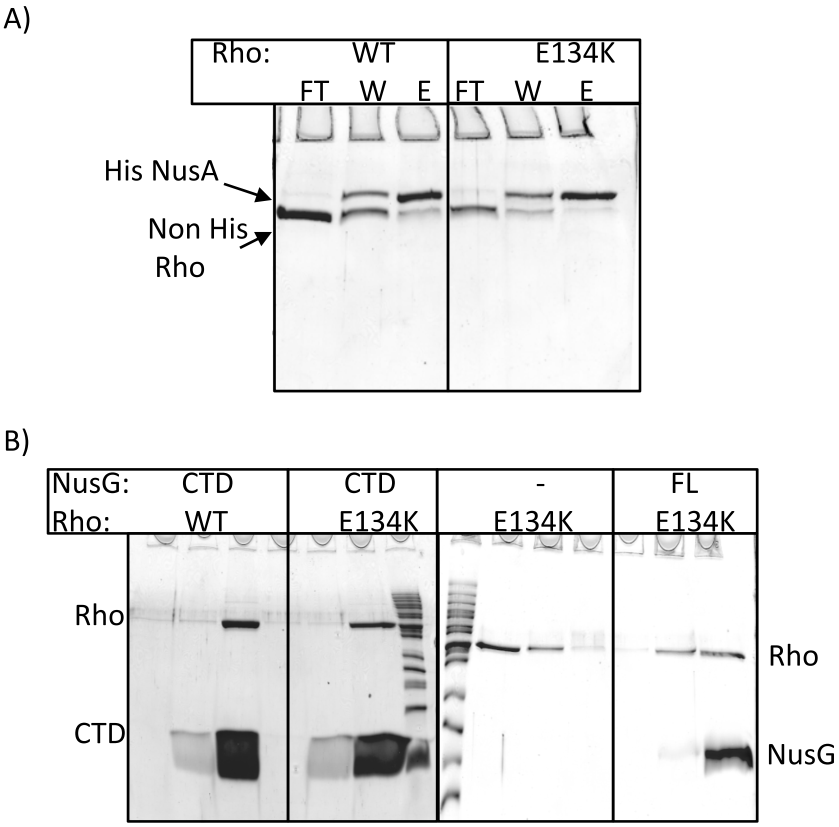

Figure S9: A) *In vitro* pull-down assays of Rho with His-tagged WT NusA. A mixture of WT or E134K Rho and WT NusA proteins was passed through Ni-NTA agarose beads. Different fractions (FT: flow-through fraction from the column; W: washed fraction E: eluted fraction from the column) were loaded onto 12% SDS-PAGE and visualized by Coomassie blue staining. Amounts of Rho and NusA used were 6 µg and 30 µg, respectively. **B)** In vitro pull-down assays of His-tagged WT NusG(Full Length, FL or NusG-CTD, CTD) with WT or E134K Rho. Different fractions with the same notations as in (A) are loaded onto 15% SDS-PAGE and viewed by Coomassie blue staining.

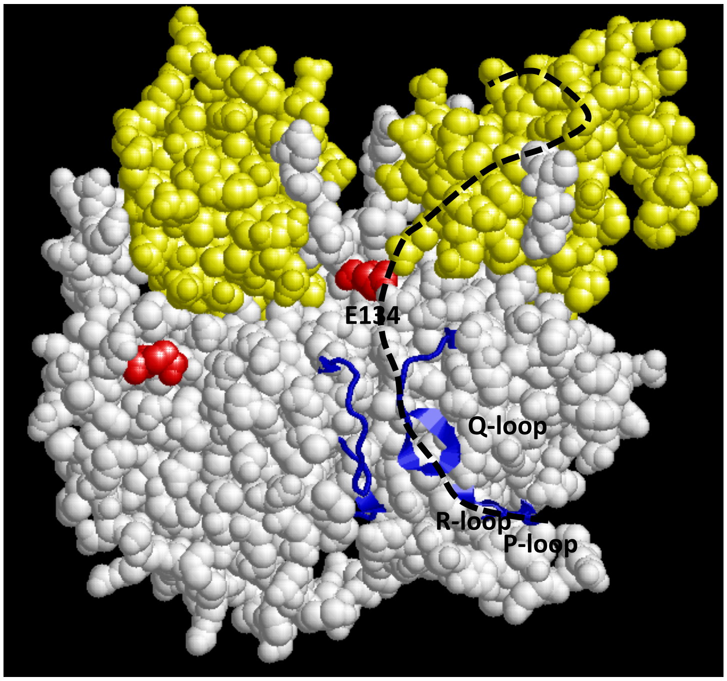

Figure S10. Location of E134 on the dimeric structure of Rho shown as red sphere. Primary RNA binding domain is in yellow and the P, Q and R-loops are in blue ribbons. The putative RNA-path through the central hole of Rho is shown in black dashed curve.

Table S1: *In vivo* antitermination defects of H-19B N mutants at the Rho-independent terminator $T_{R'}$

H-19B N alleles	***P_{lac}-nutR-$T_{R'}$-LacZYA β–galactosidase activity (A.U.)***		
	+ Ter [a]	***-Ter [b]***	***%RT***
WT	1227±7	2916±89	42.1
NΔ96-127	347±15	2745±372	12.6
NΔ101-127	409±5	2485±153	16.5
NΔ106-127	633±13	3006±116	21.1
NΔ111-127	498±43	2851±112	17.4
NΔ116-127	461±14	2622±51	17.6
NΔ121-127	1258±21	2912±130	43.2
R18P	56±2	2201±150	2.5

The above strains were transformed with the plasmids bearing different WT and mutant H-19B *N* genes. The ratio of β-galactosidase values in the presence and absence of terminator gives the efficiency of terminator read-through (%RT). The averages of 4 to 5 independent measurements are shown. CTD deletions are shown to be significantly defective on hairpin-dependent terminator indicating that the N-mediated RNAP modification plays the major role.

[a] Strain RS1018.

[b] Strain RS445.

Table S2: Termination activity of the suppressor mutants of Rho in the absence of H-19B N.

Rho alleles	*tR1*-lacZYA[a] β−galactosidase activity (A.U.)			*tR1-trpt'*-lacZYA[b] β−galactosidase activity (A.U.)		
	+Ter (RS734)	-Ter(RS445)	%RT	+Ter (RS1017)	-Ter (RS445)	%RT
WT	70 ± 6	1420 ±25	5.0	15±0.6	705±22	1.1
E134K	298 ±5	705 ±22	42.0	84±3	705±22	12

The above strains were transformed with the plasmids bearing different WT and mutants of *Rho* genes. The chromosomal copy of *Rho* was deleted by P1 transduction. The ratio of β-galactosidase values in the presence and absence of terminator gives the efficiency of terminator read-through (%RT). The average of 4 to 5 independent measurements is shown.

[a] Strains RS734 and RS445 with plasmids bearing *rho* alleles.

[b] Strains RS1017 and RS445 with plasmids bearing *rho* alleles.

Table S3: *In vivo antitermination in the presence of NusG mutants defective for Rho-binding.*

NusG alleles	***P_{lac}-nutR/tR1-lacZYA*** [a] ***β–galactosidase activities (A.U.)***			***P_{lac}-nutR/tR1-trpt'-lacZYA*** [b] ***β–galactosidase activities (A.U.)***			***Efficiency of phage platting (E.O.P.)***	
	+Ter	***-Ter***	***%RT***	***+Ter***	***-Ter***	***%RT***	***H-19B***	***λ***
WT	1226±21	3134±77	39.1	1103±42	3134±77	35.2	(1)	(1)
G146D	1039±33	2775±163	37.4	926±49	2775±163	33.4	1	1
L158Q	973±25	2408±72	40.4	795±69	2408±72	33.0	1	1

The above strains were transformed with plasmid bearing WT H-19B N gene. They were then transformed with the plasmids bearing different WT and mutants of *nusG* genes. The chromosomal copy of *nusG* was deleted by P1 transduction. The ratio of β-galactosidase values in the presence and absence of terminator gives the efficiency of terminator read-through (%RT). The averages of 4 to 5 independent measurements are shown.

Relative efficiency of plaque formation (E.O.P.) of H-19B and λC1857 phages are shown in columns 4 and 5, respectively. The number of plaques obtained with the WT enzyme was set as 1.

[a] Strains RS734 (+ter) and RS445 (-ter) with WT H-19B N and with plasmids bearing *nusG* alleles.

[b] Strains RS1017 (+ter) and RS445 (-ter) with WT H-19B N and with plasmids bearing *nusG* alleles.

Table S4. Strains, plasmids, phages and oligos.

Strains	Description	Reference
GJ3161 (RS257)	MC4100 *galEp3*	1
GJ5147 (RS734)	MC4100 *galEp3*, P_{lac}– H-19B *nutR-tR1- lacZYA*	J. Gowrishankar
RS99	K9774 (K37 strain with H-19BStx:: Cam^R lysogen).	David Friedman.
RS445	GJ3161, λRS88 lysogen carrying P_{lac} *–lacZYA*	6
RS764	MG1655 Δ*rac::* Cam^R	Max Gottesman
RS791	MG1655 Δ*rac::* Cam^R , Δ*rho::* Kan^R with pHYD1201 Amp^R	This study
RS862	MG1655 Δ*rac::* Tet^R	J. Gowrishankar
RS865	MG1655 Δ*rac::* Tet ^{R} Δ*NusG::*Kan^RwithpHYD751, Amp^R	This study
RS940	GJ3161, λRS88 lysogen carrying P_{lac} *–lacZYA, rpoC*120 btuB::Tn10(Tet^R, ts)	This study
RS941	MC4100 *galEp3*, P_{lac}– H-19B *nutR- lac ZYA, rpoC*120 btuB::Tn10(Tet^R, ts)	This study
RS1017	MC4100 *galEp3,* λRS45 lysogen carrying P_{lac}– H-19B *nutR-tR1- trpt'-lac ZYA*	This study
RS1018	MC4100 *galEp3,* λRS45 lysogen carrying P_{lac}– H-19B *nutR-tR1- tR' -lac ZYA*	This study
RS1019	MC4100 *galEp3,* λRS45 lysogen carrying P_{lac}–- λ*nutR*-λ*tR1 –lac ZYA*	This study
RS1029	MC4100 *galEp3,* λRS45 lysogen carrying P_{lac}– H-19B *nutR-tR1- trpt'-lac ZYA, , rpoC*120 btuB::Tn10(Tet^R, ts)	This study
RS1038	MC4100 *galEp3,* λRS45 lysogen carrying P_{lac}- *trpt'-lac ZYA*	This study
XL1-Red	*endA1 gyrA96 thi-1 hsdR17 supE44 relA1 lac mutD5 mutS mutT* Tn*10* (Tet^R)	Stratagene
**Phages**		
λRS45, λ*c*I857		J. Gowrishankar
H-19B (*Stx*:: Cam)		David Friedman
**Plasmids**		
pK8601	pGB2 with plac- H-19B N, $Spec^R$.	2
pK8628	pTL61T with P_{lac} – H-19B *nutR-tR1 -lacZYA* fusion, Amp^R	4
pK8641	pTL61T with P_{lac} – H-19B *nutR*-TR'T1T2-lacZYA fusion. TR'T1T2 is a triple terminator cassette, Amp^R.	2
pHYD751	2. 1-kb chromosomal fragment carrying nusG+ cloned into the EcoRI/SalI sites of pAM34 (pMB1; Amp^R)	1
pHYD1201	3.3 kb HindIII-SalI fragment carrying rho+ sub-cloned from pHYD567 into HindIII-SalI sites of pAM34 (pMB1; IPTG dependent replicon, Amp^R)	1
pRS22	pTL61T with pT7A1– H-19B *nutR*-tR'-T1T2-*lac*ZYA, Amp^R.	4
pRS96	WT *rho* cloned at NdeI/XhoI site of pET21b, His-tag at C-terminal, Amp^R	21
RS102	trpt' cloned at HindIII/BamHI sites of pK8641, Amp^R	Pani, B. 2009. PhD thesis
pRS106	T7A1 promoter cloned at EcoRI/HindIII sites of pRS102, Amp^R	3
pRS256	pGB2 with plac- λN, $Spec^R$	This study
pRS385	pRS25 with T7A1-*nutR*-*lac*O-tR' fusion, Amp^R	5
pRS431	pTL61T with P_{lac} – *lacZYA* by deletion of H-19B *nutR- tR1* between HindIII and BamHI sites from pK8628, Amp^R	6
pRS580	ARM mutant of H-19B N cloned at ClaI/BamHI sites of pK8601, $Spec^R$	This study
pRS604	pTL61T with pT7A1-Lambda *nutR*-tR1-T1T2-lacZYA, Amp^R.	This study
pRS649	WT *rho* with its own promoter cloned at HindIII/SacI sites of pCL1920, $Spec^R$, $Strep^R$	This study
pRS668	H-19B N cloned at EcoRI/PstI sites of pBR322, Tet^R	This study
pRS781	pK8601 H-19B N R15C, $Spec^R$	This study
pRS782	pK8601 H-19B N Δ 88-127, $Spec^R$	This study

pRS783	pK8601 H-19B N R3H, $Spec^R$	This study
pRS784	pK8601 H-19B N Δ 79-127, $Spec^R$	This study
pRS786	*rho* P103L by SDM of pRS96, Amp^R	This study
pRS793	pK8601 H-19B N S11F, $Spec^R$	This study
pRS892	pK8601 H-19B N R18P, $Spec^R$	This study
pRS895	pK8601 H-19B N R15P, $Spec^R$	This study
pRS896	pK8601 H-19B N R15P, H99Q, $Spec^R$	This study
pRS911	H-19B N Δ 101-127 cloned at ClaI/BamHI sites of pK8601, $Spec^R$	This study
pRS912	H-19B N Δ 116-127 cloned at ClaI/BamHI sites of pK8601, $Spec^R$	This study
pRS913	H-19B N Δ 121-127 cloned at ClaI/BamHI sites of pK8601, $Spec^R$	This study
pRS914	H-19B N Δ 106-127 cloned at ClaI/BamHI sites of pK8601, $Spec^R$	This study
pRS927	pK8601– H-19B N Δ 78-127, $Spec^R$	This study
pRS932	H-19B N Δ 111-127 cloned at ClaI/BamHI sites of pK8601, $Spec^R$	This study
pRS939	H-19B N Δ 96-127 cloned at ClaI/BamHI sites of pK8601, $Spec^R$	This study
pRS965	WT *rho* cloned at NdeI/XhoI site of pET21b, HMK tag and His-tag at C-terminal, Amp^R	This study
pRS968	Lambda N cloned at ClaI/BamHI site of pK8601, $Spec^R$	This study
pRS969	Lambda N Δ(73-107) cloned at ClaI/BamHI site of pK8601, $Spec^R$	This study
pRS990	pTL61T with P_{Lac}– H-19B *nutR-tR1- tR’ -lac ZYA,* Amp^R	This study
pRS992	pTL61T with P_{lac}–H-19B *nutR-tR1- trpt’-lac ZYA,* Amp^R	This study
pRS1074	Lambda N Δ 81-107 cloned at ClaI/BamHI sites of pK8601, $Spec^R$	This study
pRS1075	Lambda N Δ 91-107 cloned at ClaI/BamHI sites of pK8601, $Spec^R$	This study
pRS1076	Lambda N Δ 101-107 cloned at ClaI/BamHI sites of pK8601, $Spec^R$	This study
pRS1092	pTL61T with pT7A1-H-19B *nutR*-tR1-trpt’-lacZYA, Amp^R	This study
pRS1161	pCL1920 rho E134K, $Spec^R$	This study
pRS1162	*rho* E134K cloned at NdeI/XhoI site of pET21b, Non His tagged, Amp^R	This study
pRS1194	*rho* E134K cloned at NdeI/XhoI site of pET21b, HMK tag and His-tag at C-terminal, Amp^R	This study
**Oligos**		
RS58	ATAAACTGCCAGGAATTGGGGATCG; FP of pTL61T (and all its derivatives like pRS106, pRS25) vector sequence.	
RS83	ATAAACTGCCAGGAATTGGGGATCG; 5’ biotinylated RS58	
RS132	TAAGGAGGTATATCGATAATGACACGCAGAACTCAG; H-19B N FP with ClaI site, to amplify from pK8601.	
RS133	GCTGCAGGTCGACGGATCCTTAGTTACTTACCCGG; H-19B N RP with BamH1 site to amplify from pK8601.	
RS177	GAATTGTGAGCGCTCACAATTCggatATATATTAACAATTACCTG; lacO fusion at 161U of trpt’ terminator.	
RS404	GAATTGTGAGCGCTCACAATTCggatGCCAGACCGCGC TGGGTAAGCG; RP with lacO in H-19B tR1 termination region, used to make Road block template.	
RS421	TTAATACGACTCACTATAGGGAGATAAGTAACACCGCTATTTTC; FP with T7 promoter fused 11 nt upstream of H-19B boxA sequence.	
RS422	TATTGGGACT CGCTTTGTCA GCG; RP 9 nucleotide downstream of H-19B boxB sequence.	
RS423	CTTAGTTGGTCAGATA TATTGGGAC; RP 25 nucleotide downstream of H-19B boxB sequence.	
RS 504	GCGGGATCCTCACTTATGTCCAAA CTC; RP with BamHI site to make H-19B N aa 1-100.	
RS505	GCGGGATCCTCAACCAGTTTCTGGTTGC; RP with BamHI site to make H-19B N aa 1-105.	
RS 506	GCGGGATCCTCAATTTGGAAGACATAC ; RP with BamHI to make H-19B N aa 1-110.	
RS507	GCGGGATCCTCACGCGTAAAGAGCTAC; RP with BamHI site to make H-	

	19B N aa 1-115.	
RS508	GCGGGATCCTCATTTCCGGTAGCCTGC; RP with BamHI site to make H-19B N aa 1-120.	
RS509	ACTTCGGATTATCCCGTGACAGG; FP of pK8601 with EcoRI site	
RS527	GCGGGATCCTCAATTCGCGTACACAATGG; RP with BamHI site to make H-19B N aa 1-95.	
RS552	GCGCATCGATATGGATGCACAAACACGCCGC; λ N FP with ClaI site.	
RS553	GCGCGGATCCCTATTGCAGGTTGCTTTCAATCTG; RP with BamHI site and stop codon to clone Lambda ΔCTD N in pK8601	
RS567	GCGCGGATCCACCCCGGTCGAACGTCAAC; trpt' FP with Bam HI site	
RS568	GCGCGGATCCATGAGAATTTAGTCAAATTAAGC; Trp t' RP with Bam HI site	
RS 600	GCGCGGATCCCTAGCGCTGATTCTTGCGC; RP with BamHI site and stop codon to clone Lambda N-80 in pK8601	
RS 601	GCGCGGATCCCTAGCCGCGTTCGCCAGGC; RP with BamHI site and stop codon to clone λ N-90 in pK8601	
RS602	GCGCGGATCCCTACTTAATTTTCTGGCGTCC; RP with BamHI site and stop codon to clone λ N-100 in pK8601	
RS662	GTGAAAATAGCGGTGTTACTTATG; antisense to *nutR boxA* of H-19B.	
RS663	CGTAGGACGAATGTCCATTGTG; antisense to *nutR spacer*of H-19B.	
RS664	GGGACT CGCTTTGTCAGCGACGTA; antisense to *nutR boxB* of H-9B.	
RS665	CCTTAGTTGGTCAGATATATTGGGAC; antisense to region immediately after *nutRboxB* of H-19B	

References:

1. Harinarayanan, R. and Gowrishankar, J. (2003) Host factor titration by chromosomal R-loops as a mechanism for runaway plasmid replication in transcription termination-defective mutants of Escherichia coli. J. Mol. Biol., 332, 31-46.
2. Neely, M. N. and Friedman, D. I. (1998). Functional and genetic analysis of regulatory regions of coliphage H-19B: location of shiga-like toxin and lysis genes suggest a role for phage functions in toxin release. Mol. Microbiol., 28, 1255-1267.
3. Pani, B., Banerjee, S., Chalissery, J., Muralimohan, A., Loganathan, R.M., Suganthan, R.B., Sen, R. (2006). Mechanism of inhibition of Rho-dependent transcription termination by bacteriophage P4 protein Psu. J. Biol. Chem., 281, 26491-26500.
4. Cheeran, A., Babu Suganthan, R., Swapna, G., Bandey, I., Achary, M. S., Nagarajaram, H. A., and Sen, R. (2005) Escherichia coli RNA polymerase mutations located near the upstream edge of an RNA: DNA hybrid and the beginning of the RNA-exit channel are defective for transcription antitermination by the N protein from lambdoid phage H-19B. J Mol Biol 352: 28–43.
5. Cheeran, A., Kolli, N., and Sen, R. (2007) The Site of Action of the Antiterminator Protein N from the Lambdoid Phage H-19B. J Biol Chem 282: 30997–31007.
6. Chalissery, J., Banerjee, S., Bandey, I., and Sen, R. (2007) Transcription termination defective mutants of Rho: role of different functions of Rho in releasing RNA from the elongation complex. J Mol Biol 371: 855-872.

Supplementary Materials and Methods(references are in accordance with the main text**)**

Materials. NTPs were purchased from GE healthcare. [γ-^{32}P]ATP (3000 Ci/mmol) and [α-^{32}P]CTP (3000 Ci/mmol) were from Jonaki, BRIT, India. Antibiotics, IPTG, lysozyme, DTT and BSA were from USB. Primers for PCR were obtained from Sigma. Restriction endonucleases, polynucleotide kinase and T4 DNA ligase were from New England Biolabs. WT *E. coli* RNA polymerase holoenzyme was purchased from Epicentre Biotechnologies. *Taq* DNA polymerase was from Roche Applied Science. Ni-NTA agarose beads were from Qiagen. Streptavidin-coated magnetic beads were from Promega. HPLC pure antisense oligos used in the foot-printing experiments were from MWG. RNAse T1 was from Ambion and RNAse H was from Epicentre. All the bacterial growth media were from Difco.

Random mutagenesis and screening of H-19B N mutants. The plasmid pK8601 with the WT H-19B N gene (16) was transformed into XL1-Red mutator strain (20). The mutagenized plasmid library was isolated and electroporated into the background strain RS734 containing the P_{lac}–*nutR-tR1–lacZYA* cassette. The transformants were platted on MacConkey-lactose plates supplemented with appropriate antibiotics. In the absence of WT H-19B, WT *rho* confers a *lac*$^-$ phenotype because of Rho-dependent transcription termination at the *tR1* terminator. Presence of WT N confers the *lac*$^+$ phenotype and the colonies appeared as red on MacConkey-lactose plates. N mutants defective for antitermination appeared as pink/white colonies on these plates. *Lac* $^-$ transformants were picked, purified three times by streaking in the same medium. Approximately 100,000 colonies were screened. The putative N mutant plasmids were isolated and re-transformed into the background strain for ensuring the mutant phenotypes and the plasmids were sequenced subsequently to confirm the mutations. This screen yielded five point mutations, R3H, S11F, R15C, R15P, R18C in the ARM region and two C-terminal deletions, Δ(78-127) and Δ(88-127). These two deletions were obtained due to the insertion of stop codons.

Screening of suppressors in Rho. pRS649 containing the WT *rho* gene was used to obtain the mutagenised *rho* plasmid library in the similar way as described above for N gene. The mutagenised Rho library was electroporated into the strain RS1017 (P_{lac}-–*nutR-tR1-trpt'–lacZYA)* containing pRS668 with WT H-19B N gene. The transformants were directly platted on MacConkey agar plates supplemented with 1% (w/v) lactose and appropriate antibiotics to get dominant Rho mutants in the presence of a WT copy in the chromosome. Alternatively, the

chromosomal copy of *rho* gene of the transformants was removed by P1 transduction, and then platted. Rho mutants that can suppress N function appeared as pink/white colonies on these plates. Desired colonies were picked, purified as described above and subsequently sequenced to confirm the mutations.

Phage growth assays. To check whether the mutant *nusG* and *rho* alleles support the growth of phage λ and H-19B, plasmid containing these mutants were transformed into the strains RS865 (for *nusG* alleles) and RS791 (for *rho* alleles). The shelter plasmids pHyd751 and pHyd1201 were knocked out during this process. Serial dilutions of the bacteriophages H-19B (obtained from strain K9774, H-19B lysogen; a gift from Dr. David Friedman) and λ*C1857* were spotted onto the lawns of respective strains. Plaques were counted after overnight incubation at 37 ^{o}C. Efficiency of plaque formation (E.O.P) was measured relative to WT, where the efficiency for WT was fixed at 1 (Figure S8 and table S3).

Construction of deletion derivatives of N. PCR primers were designed to amplify *N* or fragments of *N* from plasmid pRS26 (for H-19B N) and pRS256 (for λ N). Forward and reverse primers contained ClaI and BamHI restriction sites, respectively, for subsequent cloning into the vector, pK8601.

Purifications of H-19B N, Rho, GreB, NusA and NusG proteins. WT, ΔCTD and ARM H-19B N, NusA and NusG proteins were purified as described earlier (16). P103L Rho was constructed by site directed mutagenesis on pRS96. E134K rho was amplified from pCL1920 using deep-vent proofreading enzyme and cloned into pET21b at NdeI/XhoI sites. Different Rho proteins were purified according to published procedure (21). Non his-tagged E134K Rho was purified by batch elution method with using 0.1 to 1M NaCl salt and passing the cell lysate over a heparin–agarose column (22). Purification of GreB was described earlier (8). HMK and His tag was at the N-terminal of NusA and purification was done using Ni-NTA beads.

Templates for in vitro transcription. Linear DNA templates for *in vitro* transcription assays were made by PCR amplification from the plasmids pRS22 (T7A1-*nutR-tR1)*, pRS1092 (T7A1-*nutR-tR1-trpt'*), pRS385 (pT7A1-H-19B *nutR/tR1*-lacO-*tR'*) and pRS106 (pT7A1-*trpt'*). When required, lac operator sequence was inserted either after *tR1* or *trpt'* terminators using a downstream primer having this sequence. In pRS385, the operator sequence is cloned in the

plasmid after the *tR1* terminator (17). 5' biotinylation of the templates were done by using the biotinylated primer RS83 and immobilization was done on streptavidin coated magnetic beads (Promega).

Association assays of Rho with the EC. Association of WT or mutant Rho (Y80C) with the stalled EC was measured by using radio-labelled HMK tagged Rho (figure S7; 23). Radio-labelling of the HMK-tag was performed by protein kinase C using [γ- ^{32}P]ATP. The template pT7A1-*nutR*-lacO-*tR'* immobilized on the magnetic beads was used. Stalled EC was formed as above either in the absence or presence of N. The free NTPs were removed by extensive washing of the beads, followed by incubation with 25 nM of [γ- ^{32}P]ATP labelled Rho in presence of 1mM AMPPNP. At different time points, half of the supernatant was taken out for the "S" lanes and the rest was used for the "S+P" lanes. The supernatant and the pellet fractions were mixed with SDS loading dye and loaded onto12% SDS PAGE. Association of Rho with EC was analyzed by exposing the gel to a Phosphor imager screen for 1 hr. and subsequently scanning using Typhoon 9200 (Amersham) followed by quantification with Image QuantTL software (figure S7).

In vitro chasing of the stalled EC. To measure the chasing efficiency of a stalled elongation complex bound to Rho in the absence and the presence of N/NusA/NusG, we used the template, pT7A1-*nutR*-lacO-*tR'*, and a stalled EC was formed at the lac operator (lacO) site in the same way as described above, both in the absence and presence of N (figures S7D, E). WT Rho in the presence of 1 mM ATP and 0.5 μM GreB was added to the stalled EC and the reactions were incubated for different time points, which was followed by addition of 1 mM IPTG and 250 μM NTPs to chase this stalled EC through the downstream *tR'* intrinsic terminator. The reaction was stopped by extracting with phenol after 5 min. of incubation at 32 °C, mixed with equal volume of formamide loading dye and loaded onto an 8% sequencing gel. GreB was added to minimize the natural arrest of the EC at the lac operator site.

Gel-shift assays. Radio-labelled RNA molecules were incubated with Rho in the transcription buffer (described above), supplemented with 10% glycerol and 1 mM AMPPNP for 10 min. at 37°C. These reaction mixtures were loaded onto gradient native PAGE (8-12%) casted in 0.5× TBE (Tris–boric acid–EDTA) buffer under running condition. Electrophoresis was also

performed in 0.5× TBE buffer in cold. Gels were then dried and analyzed by Phosphorimager (Fuji).
